# DDIT3 deficiency ameliorates systemic lupus erythematosus by regulating B cell activation and differentiation

**DOI:** 10.1093/lifemedi/lnaf009

**Published:** 2025-03-03

**Authors:** Xin Dai, Jiali Yu, Yunfei Zhang, Zhiming Wang, Yunyan Dai, Ying Hu, Xiaocui Wang, Binbin Tian, Minhui Wu, Hao Chen, Ruigao Song, Dan Ma, Cong-yi Wang, Dawei Ye, Ziliang Zheng, Liyun Zhang, Jing Luo, Yukai Jing

**Affiliations:** Institute of Clinical Medicine, Central People’s Hospital of Zhanjiang, Zhanjiang 524045, China; Department of Rheumatology, The Second Hospital of Shanxi Medical University, Taiyuan 030009, China; Department of Clinical Laboratory, Third Hospital of Shanxi Medical University, Shanxi Bethune Hospital, Shanxi Academy of Medical Sciences, Tongji Shanxi Hospital, Taiyuan 040030, China; Department of General Surgery, Third Hospital of Shanxi Medical University, Shanxi Bethune Hospital, Shanxi Academy of Medical Sciences, Tongji Shanxi Hospital, Taiyuan 040030, China; Department of General Surgery, Third Hospital of Shanxi Medical University, Shanxi Bethune Hospital, Shanxi Academy of Medical Sciences, Tongji Shanxi Hospital, Taiyuan 040030, China; Department of Clinical Laboratory, Third Hospital of Shanxi Medical University, Shanxi Bethune Hospital, Shanxi Academy of Medical Sciences, Tongji Shanxi Hospital, Taiyuan 040030, China; Department of Clinical Laboratory, Third Hospital of Shanxi Medical University, Shanxi Bethune Hospital, Shanxi Academy of Medical Sciences, Tongji Shanxi Hospital, Taiyuan 040030, China; Department of Critical Care Medicine, Central People’s Hospital of Zhanjiang, Zhanjiang 524045, China; Institute of Clinical Medicine, Central People’s Hospital of Zhanjiang, Zhanjiang 524045, China; Key Laboratory of Biomedical Information Engineering of Ministry of Education, Biomedical Informatics & Genomics Center, School of Life Science and Technology, Xi’an Jiaotong University, Xi’an 710049, China; Center of Reproductive Medicine, Shanxi Bethune Hospital, Shanxi Academy of Medical Sciences, Tongji Shanxi Hospital, Third Hospital of Shanxi Medical University, Taiyuan 030032, China; Department of Rheumatology, Shanxi Bethune Hospital, Shanxi Academy of Medical Sciences, Tongji Shanxi Hospital, Third Hospital of Shanxi Medical University, Taiyuan 030032, China; Department of Respiratory and Critical Care Medicine, the Center for Biomedical Research, NHC Key Laboratory of Respiratory Diseases, Tongji Hospital, Tongji Medical College, Huazhong University of Science and Technology, Wuhan 430030, China; Cancer Center, Tongji Hospital, Tongji Medical College, Huazhong University of Science and Technology, Wuhan 430030, China; Laboratory of Molecular Imaging, Fifth hospital of Shanxi Medical University (Shanxi Provincial People’s Hospital), Taiyuan 030032, China; Department of Rheumatology, Shanxi Bethune Hospital, Shanxi Academy of Medical Sciences, Tongji Shanxi Hospital, Third Hospital of Shanxi Medical University, Taiyuan 030032, China; Department of Rheumatology, The Second Hospital of Shanxi Medical University, Taiyuan 030009, China; Department of Clinical Laboratory, Shanxi Province Clinical Research Center for Dermatologic and Immunologic Diseases (Rheumatic diseases), Shanxi Bethune Hospital, Shanxi Academy of Medical Sciences, Tongji Shanxi Hospital, Third Hospital of Shanxi Medical University, Taiyuan 030032, China; Shanxi Academy of Advanced Research and Innovation, Taiyuan 030032, China

**Keywords:** systemic lupus erythematosus, B cells, DDIT3, PI3K

## Abstract

Systemic lupus erythematosus (SLE) is characterized by the overproduction of autoantibodies, and B cells are considered to be the primary cells involved in the development of SLE. Studies have shown that DNA damage responses play a role in B cell activity in SLE. However, the exact role of DNA damage-induced transcript 3 (DDIT3) in humoral immune response and SLE pathogenesis remains unknown. We observed increased expression of DDIT3 in B cells of SLE patients and this expression was positively correlated with disease activity. In DDIT3-knockout mice, we observed disturbances in B cell development and differentiation, inhibition of B cell activation, and BCR signaling. In addition, DDIT3 deficiency leads to a reduction in T-cell-dependent humoral immune responses. Mechanistically, we found that DDIT3 promotes the transcription and expression of *Itgad*, which enhances PI3K signaling and B cell activation. Finally, we found that DDIT3 deficiency attenuated lupus autoimmunity and reduced germinal center responses. In conclusion, our study reveals for the first time the role of DDIT3 in adaptive immune responses, especially in B cell homeostasis, B cell activation, BCR signaling, and B cell function. These findings provide a new potential target for therapeutic intervention in SLE.

## Introduction

Systemic lupus erythematosus (SLE) is a common autoimmune disease that mainly affects women. It is characterized by the deposition of large amounts of autoantibodies and antigen–antibody complexes, leading to multiple organ involvement. SLE causes serious harm to vital organs, such as the kidneys, blood, and nervous system, often resulting in irreversible damage that accumulates over time and ultimately leads to patient death [1, 2]. However, the exact pathogenesis and etiology of SLE remain incompletely elucidated.

The pathogenesis of SLE is characterized by the production of large amounts of autoantibodies, and B cells, the primary effector cells for antibody production, are considered to be the key immune cells involved in the development of SLE immunological abnormalities [2]. Current studies suggest that abnormalities in B cell development and differentiation, as well as dysregulation of the B cell receptor (BCR) signaling pathway, are involved in the pathogenesis and immunological manifestations of SLE [3–5]. In the peripheral circulation, mature B cells are activated by self-antigens or exogenous antigens, resulting in the formation of germinal center B cells (GCB) within the germinal center. These GCBs further differentiate into memory B cells (MBCs) and plasma cells, which eventually produce specific antibodies [6, 7]. Spontaneously reactive germinal centers contain auto-reactive B cells that produce pathological autoantibodies, thus contributing to the development of autoimmunity [8]. Furthermore, B cell differentiation, development, and function are regulated by BCR signals. Studies have shown that in SLE and lupus-like mouse models, BCR signaling is disrupted leading to an enhanced reactive state that promotes the onset and progression of SLE disease [9–12]. Therefore, correcting the abnormal development and differentiation of B cells, as well as addressing the BCR signaling and B cell function in SLE, is essential to address the root causes of SLE onset and progression.

The DNA damage responses (DDR) are a dynamic aspect of the development and function of the immune system. DDR is critical for the gene rearrangement, which is necessary for the formation of lymphocyte antigen receptors, antibody production, maturation, and rapid division [13, 14]. Previous studies have indicated that DDR underlies B-cell dysfunction in SLE [15]. DDR induces the upregulation of DNA damage-inducible transcript 3 (DDIT3) expression [16]. As a member of the CCAAT/enhancer-binding protein (C/EBP) family, DDIT3 regulates the expression of target genes by forming homologous dimers or binding to other C/EBP family members to form heterodimers [17]. Recent research has discovered that DDIT3 plays a significant role in innate immune response and viral infections [18–20]. However, the function of DDIT3 in adaptive immune responses, particularly in B cells, remains unclear. Studies on childhood SLE have found that DDIT3 expression is significantly elevated and positively correlates with MBCs [21]. This suggests that DDIT3 may be involved in the pathogenesis of SLE.

In this study, we found for the first time that DDIT3 expression was increased in SLE B cells and positively correlated with disease activity. DDIT3 knockout (KO) mice were used to investigate the role of DDIT3 in B-cell development and differentiation, B cell activation, BCR signaling, and T-cell-dependent humoral immune responses. We found that DDIT3 KO mice showed increased proportions of pre-pro-B cells and late pre-B cells, decreased proportions of early pre-B cells, and recirculating mature B cells in bone marrow (BM). DDIT3 KO mice showed significant reductions in splenic GCB cells and marginal zone (MZ) B cells. In addition, B cells from DDIT3 KO mice showed decreased levels of phosphorylation of proximal BCR signaling, BLNK and Syk, and distal signaling of PI3K–Akt–mTORC1 following BCR activation. Furthermore, DDIT3 deficiency suppressed T-cell-dependent humoral immune responses and ameliorated lupus symptoms in the Bm12-induced lupus model. Mechanistically, DDIT3 positively regulates *Itgad* transcription and expression, which enhances actin cytoskeletal reorganization and promotes B cell activation and PI3K signaling. In conclusion, our study identifies a novel role of DDIT3 in B cells and SLE pathogenesis, which provides a novel target for SLE treatment.

## Results

### DDIT3 expression is increased in B cells from SLE and positively correlated with the disease activity

**Figure 1.**
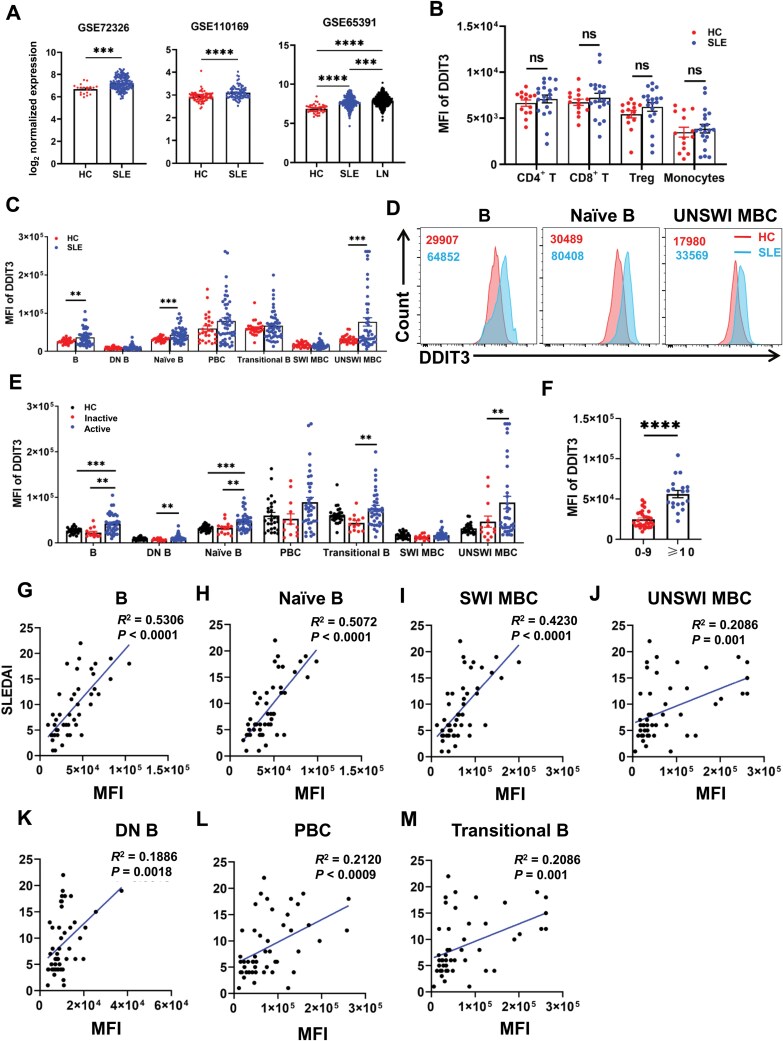
**DDIT3 was highly expressed in SLE B cells and positively correlated with SLEDAI.** (A) log_2_ fold-change of normalized RNA abundance for comparisons between groups from the dataset GSE72326 (HC, *n* = 20; SLE, *n* = 159), GSE110169 (HC, *n* = 77; SLE, *n* = 82), and GSE65391 (HC, *n* = 41; SLE, *n* = 245; LN, *n* = 667). (B) Statistical analysis of DDIT3 expression in T cell subpopulations and monocytes from SLE patients (*n* = 20) and healthy donors (*n* = 14). (C) Statistical analysis of DDIT3 expression in B cell subpopulations from SLE patients (*n* = 49) and healthy donors (*n* = 27). (D) Representation of DDIT3 fluorescence histograms in total B cells, naïve B cells, and unswitched MBCs from SLE patients and healthy donors. (E) Statistical analysis of DDIT3 expression in B cell subpopulations from active SLE patients (*n* = 36), inactive SLE patients (*n* = 13), and healthy donors (*n* = 27). (F) Statistical analysis of DDIT3 expression in B cells from severe SLE patients (*n* = 19) and non-severe SLE patients (*n* = 30). (G–M) Correlation analysis of DDIT3 expression and SLEDAI in different B cell subpopulations from SLE patients (*n* = 49). ns = no significance, **P < 0.01, ***P < 0.001, ****P < 0.0001, unpaired student’s *t*-test and one-way ANOVA.

To investigate the role of DDIT3 in the pathogenesis of SLE, we first analyzed the expression of DDIT3 in the dataset GSE72326 and GSE110169 and found that the expression of DDIT3 in SLE was significantly higher than that in healthy controls (HC) (Fig. 1A). In addition, we also found that DDIT3 expression in lupus nephritis (LN) in the dataset GSE65391 was significantly higher than that in SLE without nephritis and HC (Fig. 1A). Next, we used flow cytometry to analyze the DDIT3 expression in immune cells in SLE patients and HC (Fig. S1) and found that there was no significant difference in the mean fluorescence intensity (MFI) of DDIT3 in CD4^+^ T cells, CD8^+^ T cells, regulatory T cells (Tregs), and monocytes between patients and HC (Fig. 1B). However, the MFI of DDIT3 in total B cells, naïve B cells, and unswitched MBCs were significantly higher in patients than that in HC (Fig. 1C and 1D). These findings suggest that DDIT3 may play a crucial role in the pathogenesis of SLE by affecting B cells rather than other lymphocytes.

To further explore the impact of high expression of DDIT3 in B cells on the disease activity of SLE, patients were categorized into two groups: inactive group (SLEDAI ≤ 4) and active group (SLEDAI ≥ 5) based on the SLEDAI-2K (SLE Disease Activity Index 2000). The expression of DDIT3 in total B cells, DN (CD27 and IgD double negative) B cells, naïve B cells, transitional B cells, and unswitched MBCs were significantly higher in active group than in the inactive group (Fig. 1E). Additionally, the MFI of DDIT3 in the severe group (SLEDAI ≥ 10) was significantly higher than that in the non-severe group (SLEDAI ≤ 9) (Fig. 1F). We further analyzed the correlation between SLEDAI and DDIT3 expression, and discovered that the MFI of DDIT3 in total B cells, naïve B cells, switched MBCs, unswitched MBCs, DN B cells, plasmablast cells (PBCs), and transitional B cells was markedly positively correlated with SLEDAI (Fig. 1G–M). Additionally, the DDIT3 gene has been identified to encode multiple alternative splicing transcript variants of two isotypes of different lengths (Fig. S2A). To identify which transcription plays a role in SLE, we detected DDIT3 in peripheral blood B cells of SLE patients by Western blot (WB). The results showed that only one transcription of DDIT3 was expressed in B cells, and it was significantly higher in patients than in HC (Fig. S2B and S2C). These data above indicate that DDIT3 may be involved in the pathogenesis of SLE by affecting B cells.

### DDIT3 deficiency disrupts the development and differentiation of B cells

**Figure 2.**
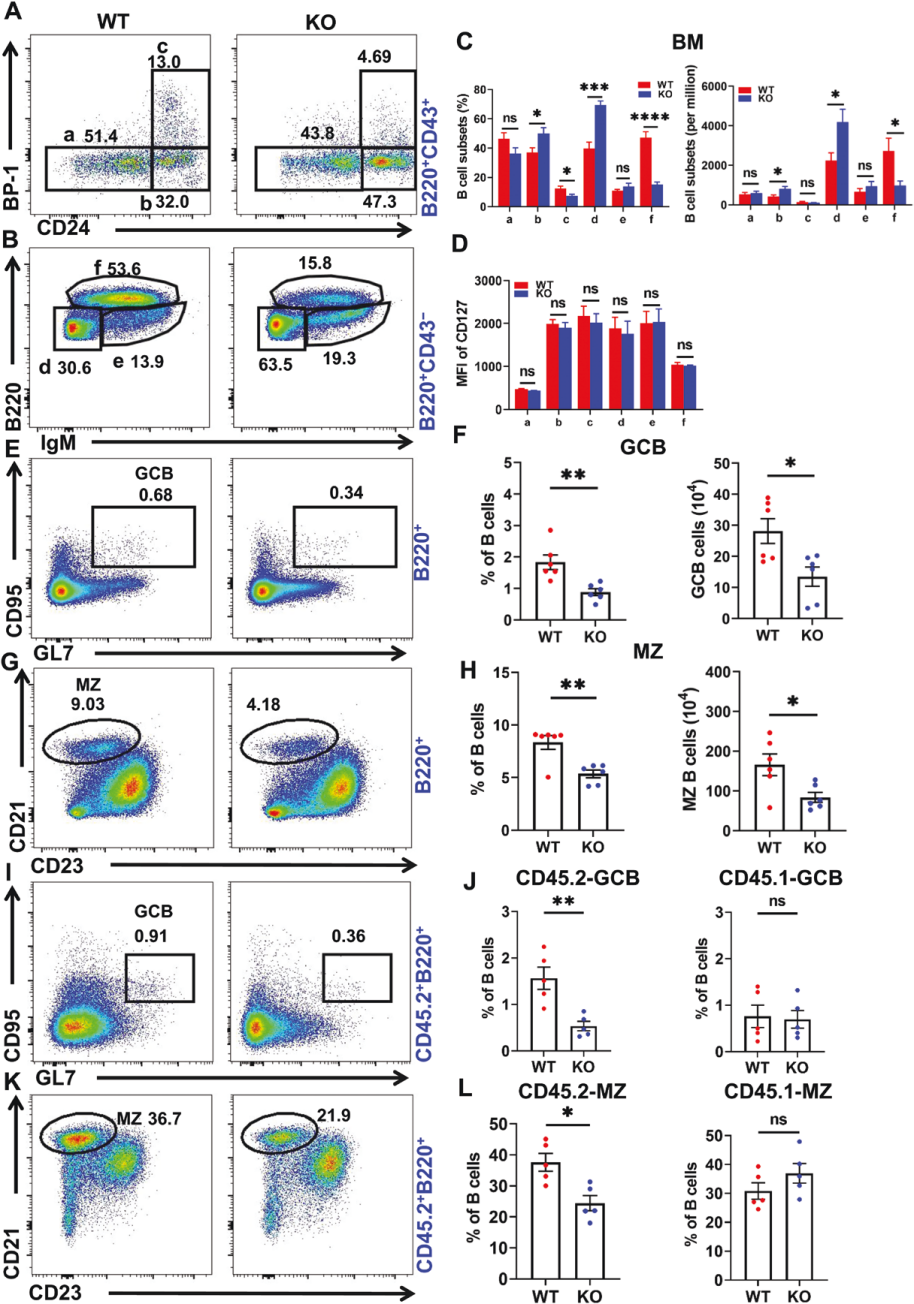
**DDIT3 is essential for the development of bone marrow B cells and differentiation of peripheral B cells.** (A–C) Representative flow diagrams and statistical analysis of pre-pro (a), pro (b), early-pre (c), late-pre (d), immature (e), and recirculating mature (f) B-cell in bone marrow from WT (*n* = 6) and DDIT3 KO mice (*n* = 6). (D) Statistical analysis of MFI of CD127 in pre-pro (a), pro (b), early-pre (c), late-pre (d), immature (e), and recirculating mature (f) B-cell in bone marrow from WT (*n* = 6) and DDIT3 KO mice (*n* = 6). (E**–**H) Representative flow diagrams and statistical analysis of splenic GC and MZ B cells in WT (*n* = 6) and DDIT3 KO (*n* = 6) mice. (I–L) Representative flow diagrams and statistical analysis of splenic GC and MZ B cells in CD45.2^+^ and CD45.1^+^ cells from chimera mice (*n* = 5). *P < 0.05; **P < 0.01; ***P < 0.001, ****P < 0.0001, unpaired student’s *t*-test.

To investigate the role of DDIT3 in B cells, we used DDIT3 KO mice (*Ddit3*^−/−^) and wild-type mice (WT, *Ddit3*^+/+^), which were genotyped by PCR, and WB (Fig. S3A and 3B). We first examined the expression of DDIT3 in B cell subpopulations using flow cytometry and found that DDIT3 was expressed in all B cell subpopulations in BM and spleen (Fig. S3C). Next, flow cytometry was used to analyze the B cell differentiation and development in the BM, spleen, and peritoneal cavities of KO and WT mice. In the BM, the numbers and percentages of pro-B cells and late pre-B cells in KO mice were significantly increased when compared to WT mice. However, compared to WT mice, the numbers and percentages of recirculating mature B cells in KO mice were significantly decreased, and the percentages of early pre-B cells in KO mice were significantly decreased while the numbers remained unchanged. There were no significant differences in the numbers and percentages of pre-pro-B cells and immature B cells between WT and KO mice (Fig. 2A–C). Since CD127 plays a crucial role in the survival, proliferation, and maturation of BM B cells [22], we also examined the changes in WT and KO mice but found no significant differences (Fig. 2D). In brief, these results indicate that DDIT3 can affect the development of BM B cells.

In the spleen, there were no significant differences in the percentage and number of follicular (FO) B cells, transition type I (T1) B cells, transition type II (T2) B cells, and isotype-switched (IS) B cells between the WT and KO mice (Fig. S4A–E). However, the percentage and number of GCB (Fig. 2E and 2F) and MZ B (Fig. 2G and 2H) were significantly lower in the KO group than that in the WT group. We then examined the proliferation and apoptosis of B cell subsets in the spleen and found no significant differences between the WT and KO mice (Fig. S4F and 4G). In addition, we examined peritoneal B1 cells in WT and KO mice and found no changes in proportions of B1a and B1b cells in these two groups (Fig. S4H–J). These results revealed that DDIT3 deficiency affects the differentiation of splenic B cell subpopulations, but not via alterations in cell proliferation and apoptosis.

To exclude the effect of DDIT3 deletion in other cells on B cell development and differentiation, we generated BM chimeras by transferring a mixture of BM cells with a 1:1 ratio of WT or DDIT3 KO mice and CD45.1 mice into irradiated CD45.1 mice. After 8 weeks, the proportions of GCB and MZ B cells in CD45.2^+^ cells from KO-CD45.1 chimera mice were lower than those in WT-CD45.1 chimera mice (Fig. 2I–L). However, the proportions of GC and MZ B cells in CD45.1^+^ cells showed no change between KO-CD45.1 chimera mice and WT-CD45.1 chimera mice (Fig. 2J and 2L). What is more, the proportions of FO, T1, T2, and IS B cells in both CD45.2^+^ cells and CD45.1^+^ cells showed no change between KO-CD45.1 chimera mice and WT-CD45.1 chimera mice (Fig. S4K). These results indicate that the decrease of GC and MZ B cells in KO mice is intrinsic to B cells.

Given the critical role of T cells in B cell differentiation, we also examined the percentage and number of T cell subpopulations in the spleen, thymus, and lymph nodes of KO and WT mice (Fig. S5A). We found no significant differences in the percentages and numbers of CD4^+^, CD8^+^, and regulatory T cells in the spleens, thymus, and LN from KO and WT mice (Fig. S5B–D). In addition, PMA and ionomycin were used to stimulate CD4^+^ T and CD8^+^ T cells from the spleen, thymus, and LN. No differences were found in the proportions of Th1 (CD44^+^IFNγ^+^CD4^+^), Th2 (CD44^+^IL4^+^CD4^+^), and Th17 (CD44^+^IL17^+^CD4^+^) cells in CD4^+^ T cells, nor in the proportions of cytotoxic T (CTL, CD44^+^IFNγ^+^CD8^+^) cells in CD8^+^ T cells between KO and WT mice (Fig. S5E–H). Hence, the absence of DDIT3 did not affect T cell development and differentiation. Taken together, our findings suggest that DDIT3 plays a crucial role in BM B cell development and peripheral B cell differentiation.

### DDIT3 deficiency weakens the B cell activation and BCR signaling

**Figure 3.**
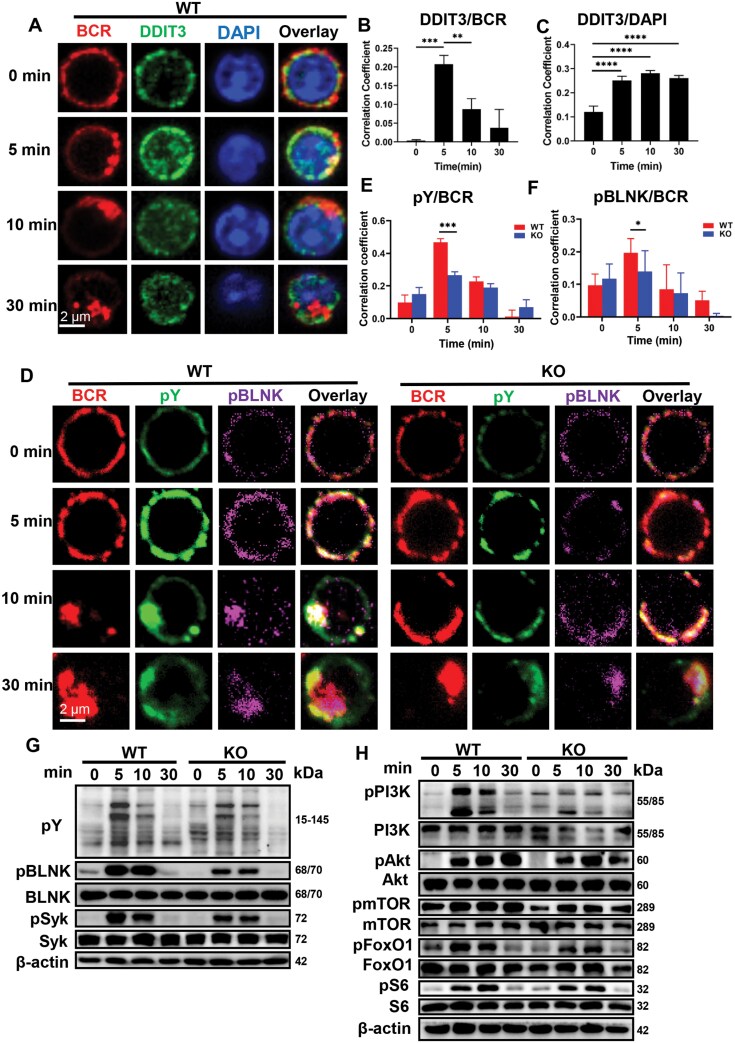
**DDIT3 positively regulates the B cell activation and BCR signaling.** Splenic B cells from WT and DDIT3 KO mice were stimulated with anti-F(ab’)^2^ goat anti-mouse IgG + IgM (10 μg/mL) for the indicated time, and the BCR signaling was analyzed by confocal and WB. (A–C) Confocal analysis of DDIT3 in splenic B cells from WT mice. Representative pictures and Pearson correlation coefficients between BCR and DDIT3/DAPI. (D–F) Confocal analysis of pY and pBLNK in splenic B cells from WT and DDIT3 KO mice. Representative plots and Pearson correlation coefficients between BCR and pY, BCR and pBLNK. Scale bar, 2 μm. Data were collected from more than 50 cells. (G, H) Immunoblot of pY, pBLNK, pSyk, pPI3K, pAkt1, pmTOR, pFoxO1, pS6, and total proteins in B cells from WT and DDIT3 KO mice. Representative plots from three independent experiments are shown. *P < 0.05; **P < 0.01; ***P < 0.001, ****P < 0.0001, unpaired student’s *t*-test and one-way ANOVA.

BCR signaling is critical for B cell development and differentiation. It has been reported that the expression of DDIT3 in B cells of the mouse spleen increases when stimulated with anti-IgM [23]. To further investigate the involvement of DDIT3 in B cell activation and BCR signaling, we stimulated splenic B cells at different time points using soluble antigenic stimulant (sAg), anti-mouse Ig (M+G) F(ab’)^2^ and found that the correlation coefficients between BCR and DDIT3 gradually increased and reached their peak after 5 min of stimulation and then gradually decreased (Fig. 3A and 3B). The correlation coefficients between DAPI and DDIT3 gradually increased after BCR stimulation (Fig. 3C). We also found that BCR internalization after 30 min of stimulation was impaired in KO B cells compared to WT B cells (Fig. 3D). These results suggest that DDIT3 plays a role in B cell activation. We then investigated the role of DDIT3 in the regulation of BCR signaling using confocal microscopy and WB. The correlation coefficients between phosphorylated tyrosine (pY, total BCR signaling), phosphorylated BLNK (pBLNK), phosphorylated Syk (pSyk), and phosphorylated PI3K (pPI3K) and BCR were significantly decreased at 5 min in KO B cells when compared to that of WT B cells (Figs. 3D–F, S6A–D). Upon sAg stimulation, the levels of pY, pBLNK, pSyk, pPI3K, and downstream signaling molecules phosphorylated Akt (pAkt), phosphorylated mTOR (pmTOR), phosphorylated S6 (pS6), and phosphorylated FoxO1 (pFoxO1) were dramatically reduced at 5 min and/or 10 min in KO B cells compared to WT B cells (Figs. 3G, 3H and S6E–K). These results above indicate that DDIT3 is involved in B cell activation and enhances BCR signaling.

### DDIT3 promotes B cell activation and BCR signaling by regulating actin reorganization

**Figure 4.**
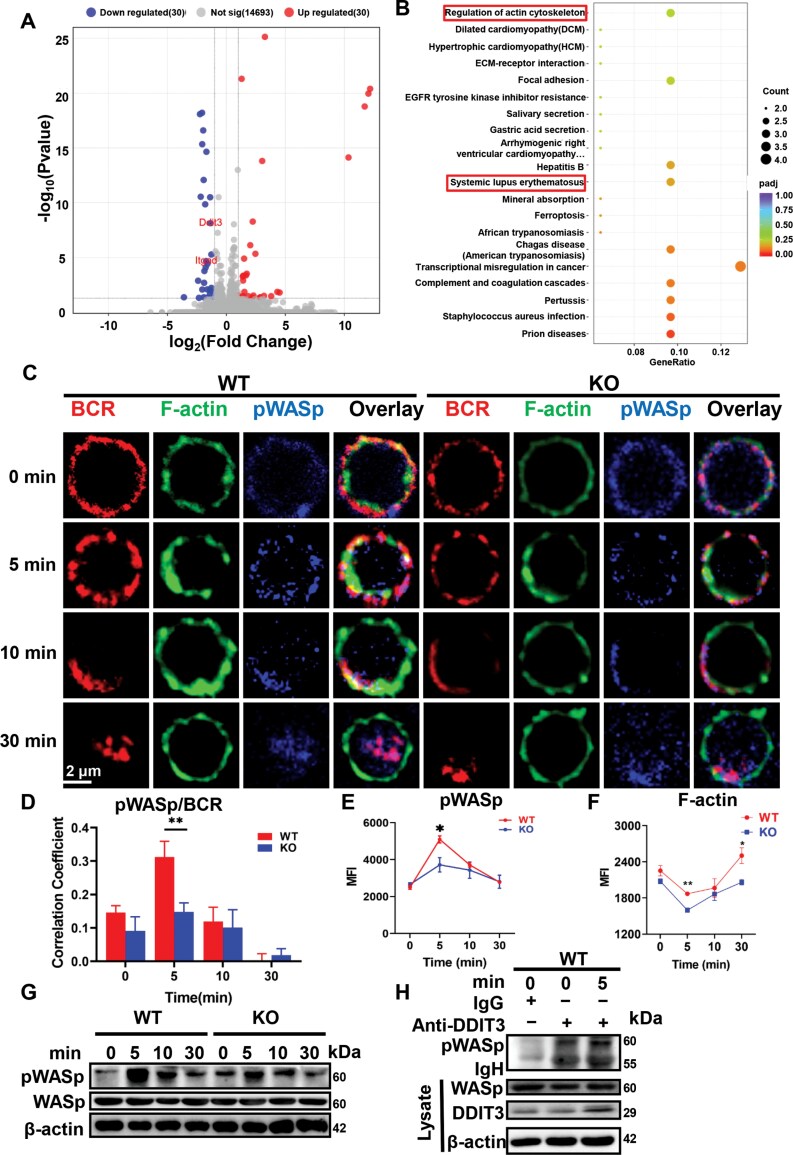
**DDIT3 is involved in actin recombination.** (A) Volcanic map analysis of RNA-seq for splenic B cells from WT (*n* = 3) and DDIT3 KO (*n* = 3) mice. (B) KEGG analysis of RNA-seq for splenic B cells from WT and DDIT3 KO mice. Splenic B cells from WT and DDIT3 KO mice were stimulated with AF594 labeled or unlabeled anti-F(ab')^2^ goat anti-mouse IgG + IgM (10 μg/mL) for the indicated time, and the BCR signaling was analyzed by confocal, phosflow cytometry, and WB. (C, D) Confocal analysis of pWASp and F-actin in splenic B cells from WT and DDIT3 KO mice. Representative pictures and Pearson correlation coefficients between BCR and pWASp. Scale bar, 2 μm. (E, F) Phosflow analysis of MFI of pWASp and F-actin in splenic B cells from WT (*n* = 3) and DDIT3 KO (*n* = 3) mice. (G) Immunoblot of pWASp and WASp in B cells from WT and DDIT3 KO mice. Representative plots from three independent experiments are shown. (H) Lysates of splenic B cells from WT mice treated with 10 µg/mL sAg for 5 min, followed by immunoprecipitation with anti-DDIT3 and immunoblot analysis of pWASp. *P < 0.05, **P < 0.01, unpaired student's *t*-test.

We further explored how DDIT3 regulates B cell activation and BCR signaling. Splenic B cells were isolated from WT and KO mice using mice B cell magnetic bead sorting kit and RNA-sequencing was conducted. 60 differential genes were screened in B cells of WT and KO mice, of which 30 were up-regulated and 30 were down-regulated (Fig. 4A; Table S3). Subsequently, KEGG and GO enrichment analysis of these differential genes indicated that DDIT3 plays a significant role in the regulation of actin cytoskeleton, SLE (Fig. 4B), humoral immune response, activation of immune response, and actin-based cell projection (Fig. S7A). Our previous study has demonstrated that the reorganization of actin during B cell activation plays a crucial role in B cell activation, BCR signaling, and antigen presentation [24, 25]. Additionally, WASp, an important actin-nucleating factor, is known to regulate actin reorganization during the early B cell activation. Therefore, we next further explored whether DDIT3 is involved in regulating actin cytoskeleton in B cells by confocal microscopy and phos-flow. We observed that the correlation coefficient between pWASp and BCR in KO B cells was noticeably reduced after 5 min of sAg stimulation compared to WT B cells (Fig. 4C and 4D). Moreover, the MFI of pWASp and F-actin in KO B cells were also significantly decreased at 5 min after sAg stimulation when compared to that of WT B cells (Fig. 4E and 4F). Consistent with these findings, the level of pWASp in KO B cells at 5 min after sAg stimulation was lower than that in WT B cells (Fig. 4G). Based on these results, we hypothesized that DDIT3 might regulate WASp phosphorylation by interacting with WASp. To demonstrate the interaction between DDIT3 and WASp, we performed co-immunoprecipitation. The results showed that DDIT3 interacted with WASp, and the pWASp increased with the activation of B cells (Fig 4H). Taken together, these results indicate that DDIT3 may promote B cell activation and BCR signaling through phosphorylating WASp and regulating actin polymerization.

### DDIT3 regulates actin reorganization by promoting Itgad transcription and expression

**Figure 5.**
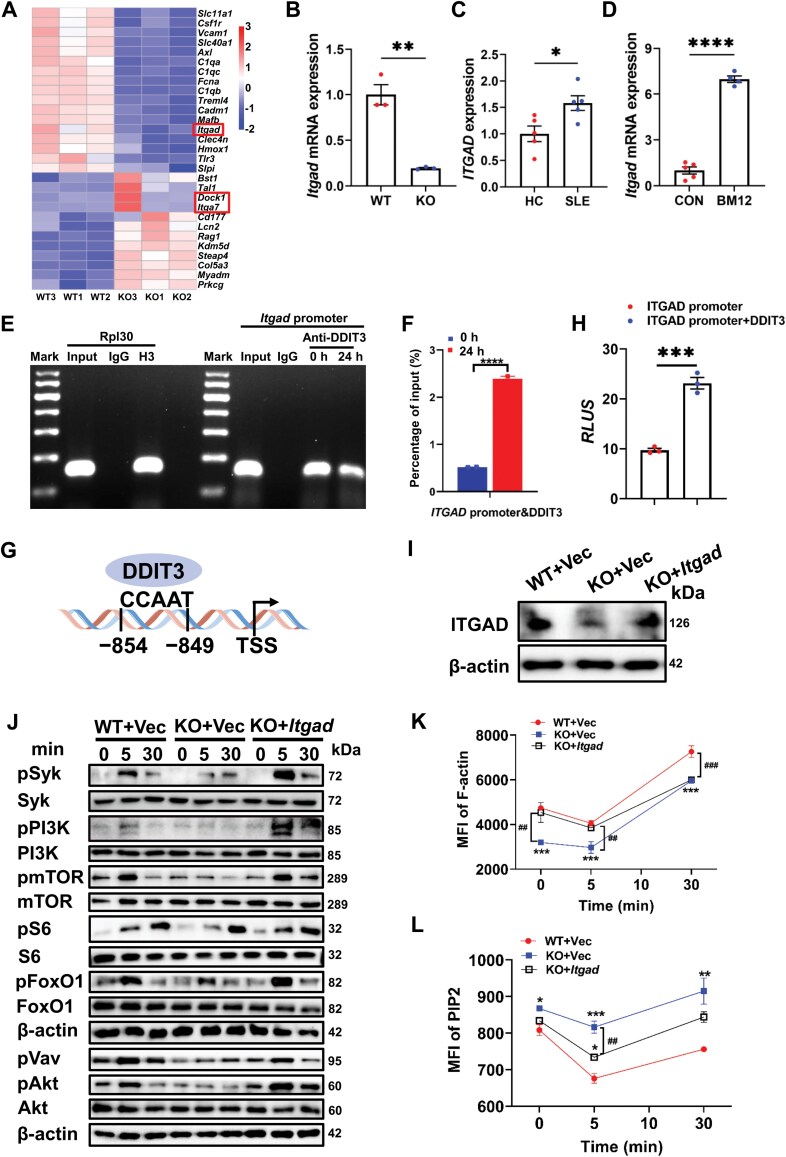
**DDIT3 positively regulates the BCR signaling pathway and actin reorganization by promoting *Itgad* transcription and expression.** (A) The heatmap of differential genes of RNA-seq for spleen B cells from WT (n = 3) and DDIT3 KO (*n* = 3) mice. (B) RT-qPCR analysis of *Itgad* mRNA in splenic B cells from WT (*n* = 3) and DDIT3 KO (*n* = 3) mice. (C) RT-qPCR analysis of *ITGAD* mRNA in B cells from SLE patients (*n* = 6) and healthy donors (*n* = 5). (D) RT-qPCR analysis of *Itgad* mRNA in splenic B cells from WT (*n* = 5) and BM12 induced-WT (*n* = 4) mice. (E, F) ChIP-qPCR analysis of DDIT3 binding to the promoter of *Itgad* in B cells after stimulation for the indicated time. (G) Transcription binding sites of *Itgad* for DDIT3. (H) Statistical analysis of relative light units (RLUs) of HEK293T cells transfected with pGL3-*Itgad* promoter plus pAd5-E1-CMV-Ddit3 and pGL3-*Itgad* plus pAd5-E1-CMV. (I) KO B cells were transfected with the plasmid overexpressing ITGAD for 48 h. Shown are immunoblot of ITGAD. (J) WB analysis of pSyk, Syk, pPI3K, PI3K, pmTOR, mTOR, pS6, S6, pFoxO1, FoxO1, pAkt, Akt, and pVav in WT, KO, and transfected KO B cells. (K, L) Phosflow analysis of F-actin and PIP2 in WT, KO, and transfected KO B cells. ^**##**^*P* < 0.01; ^**###**^*P* < 0.001; *P < 0.05; **P < 0.01; ***P < 0.001, ****P < 0.0001, unpaired student’s *t*-test and one-way ANOVA.

We further investigated how DDIT3 regulates the actin cytoskeleton by verifying the expression of three differential genes in the pathway of “Regulation of actin cytoskeleton”, *Itgad*, *Dock1*, and *Itga7* (Fig. 5A) by qPCR. Consistent with the sequencing results, *Itgad* expression was significantly decreased in KO mice (Fig. 5B), while no significant differences in *Dock1* and *Itga7* expression were found between WT and KO mice (Fig. S7B and S7C). Moreover, we extracted RNA from B cells of SLE patients and healthy controls for qPCR validation, confirming that *ITGAD* mRNA levels were higher in patients than in HC (Fig. 5C). The expression of *Itgad* in splenic B cells from the BM12-induced lupus model mice was also significantly increased when compared to control mice (Fig. 5D). To investigate whether DDIT3 regulates the transcription of *Itgad*, we conducted chromatin immunoprecipitation (ChIP)-qPCR and dual luciferase reporter assays. We found that DDIT3 could bind to the *Itgad* promoter (Fig. 5E and 5F) at the indicated binding site (Fig. 5G). Additionally, in the dual luciferase assay, the fluorescence intensity of HEK293T cells transfected with pGL3-*Itgad* promoter plus pAd5-E1-CMV-Ddit3 was significantly higher than that of cells transfected with pGL3-*Itgad* promoter plus pAd5-E1-CMV (Fig. 5H). These results above verified that DDIT3 positively regulates *Itgad* transcription and expression.

β2-integrins have been reported to enhance actin polymerization and stimulate PI3K in human neutrophils [26–28], leading to a decrease in PtdIns [4, 5]P_2_ (PIP2) levels and an increase in PtdIns [3–5]P_3_ (PIP3) levels. They can also activate downstream signaling molecules such as Src family tyrosine kinases, Syk, and Vav in T cells and B cells [29]. Therefore, we constructed a plasmid that overexpressed ITGAD and transfected it into KO B cells. The expression of ITGAD was significantly increased in the KO B cells after transfection (Fig. 5I). Next, we examined the levels of ITGAD downstream signaling molecules through WB. Upon activation with sAg, KO B cells transfected with the plasmid overexpressing ITGAD exhibited restored or increased levels of pSyk, pVav, pPI3K, as well as its downstream signaling molecules pAkt, pmTOR, pS6, and pFoxO1 (Fig. 5J). Furthermore, we examined F-actin levels using phosflow and found that the MFI of F-actin was significantly higher in KO B cells overexpressing ITGAD than in KO B cells at 0 and 5 min, with levels comparable to those in WT B cells after 0 and 5 min of B cell activation (Fig. 5K). Meanwhile, we measured the levels of PIP2 using phosflow and found that KO B cells showed significantly higher levels of PIP2 at 0, 5, and 30 min compared to WT B cells (Fig. 5L). The MFI of PIP2 in KO B cells overexpressing ITGAD was significantly decreased at 5 min and partially restored at 30 min compared to KO B cells (Fig. 5L). These data suggest that DDIT3 regulates B cell activation, BCR signaling, and actin reorganization by promoting *Itgad* transcription and expression.

### DDIT3 deficiency reduces humoral immune response and impairs germinal center response

**Figure 6.**
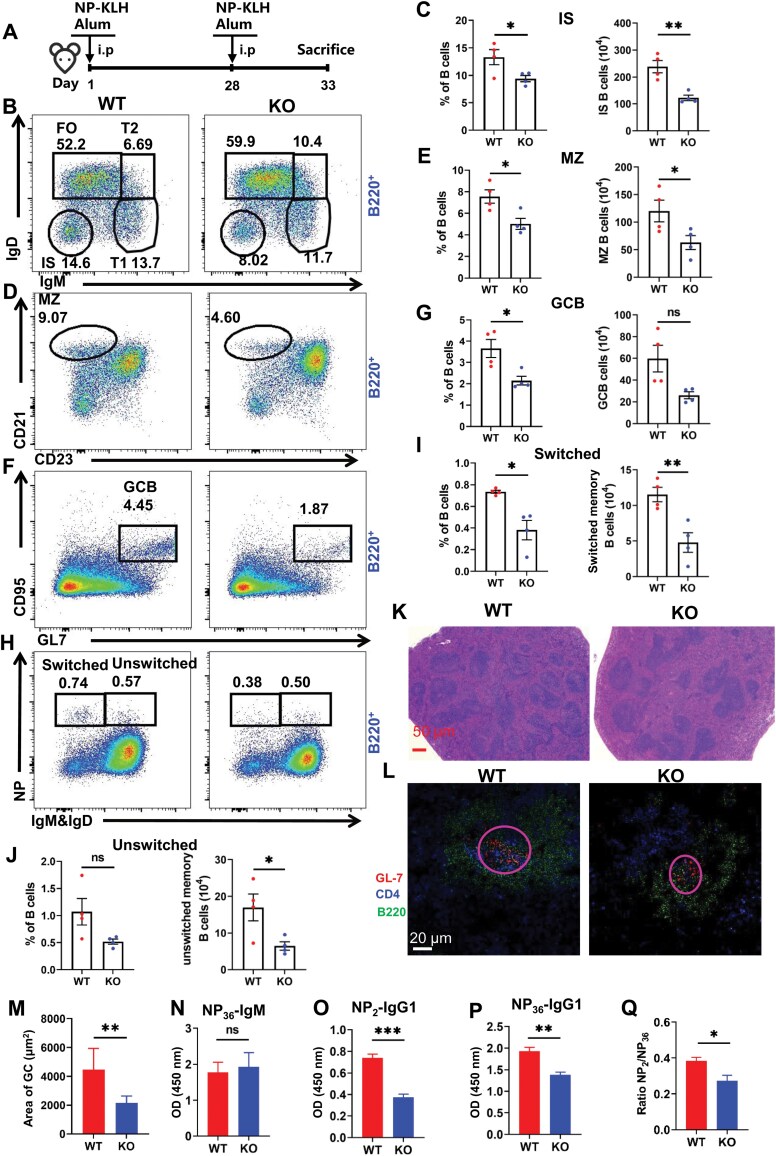
**DDIT3 deficiency reduces the T-dependent immune response.** (A) WT (*n* = 4) and DDIT3 KO (*n* = 4) mice aged 8–10 weeks were immunized by intraperitoneal injection with 40 μg NP-KLH in adjuvant on day 1 and 28, and on day 33, mice were sacrificed. (B–J) Representative flow diagrams and statistical analysis of FO, T1, T2, IS, MZ, and GC B cells, as well as switched MBC and unswitched MBC in spleens from WT and DDIT3 KO mice. (K) H&E analysis of spleens from immunized WT and DDIT3 KO mice. Scale bar, 50 μm. (L) Immunofluorescence staining of splenic sections from immunized WT and DDIT3 KO mice. Shown are representative GCs using anti-CD4 (blue), anti-B220 (green), and anti-GL-7 (red). Scale bar, 20 μm. (M) Quantification of the area of GCs from WT (*n* = 3) and DDIT3 KO (*n* = 3) mice. (N–Q) ELISA analysis of NP_36_-IgM, NP_2_-IgG1, NP_36_-IgG1, and ratio of NP_2_-IgG1/NP_36_-IgG1 in serum from immunized WT (*n* = 4) and DDIT3 KO (*n* = 4) mice. ns = no significance, *P < 0.05; **P < 0.01; ***P < 0.001, unpaired student’s *t*-test.

To investigate the impact of DDIT3 on B cell-mediated humoral immune response, we administered WT and KO mice with thymus-dependent antigen (NP-KLH) (Fig. 6A). Firstly, 33 days later after the first immunization, we analyzed splenic lymphocytes by flow cytometry and found that the frequencies and numbers of IS, MZ, GCB, and NP-specific switched MBCs were visibly reduced in KO mice compared to WT mice (Fig. 6B–J). And, the numbers of NP-specific unswitched MBCs and T1 B cells were diminished but the frequencies were unchanged (Figs. 6H, 6J and S8A). However, the frequencies and numbers of FO B cells, T2 B cells, PBC, PC, follicular helper T cells, and total CD4^+^ T cells were not significantly changed (Fig. S8B–K).

What’s more, we examined follicles and germinal centers in the spleens of WT and KO mice using H&E staining and confocal microscopy, which showed that KO mice had fewer and smaller follicles (Fig. 6K) and smaller GC areas (Fig. 6L and 6M) compared to WT mice. Furthermore, although there was no change in the frequencies of NP^+^GCB between WT and KO mice, the number of NP^+^GCB was significantly decreased in KO mice when compared to WT mice (Fig. S8L and S8M). These results suggest that DDIT3 deficiency impairs germinal center responses.

Finally, we measured NP-specific antibodies, including anti-NP_36_-IgM, anti-NP_2_-IgG1 (high affinity), and anti-NP_36_-IgG1 (low affinity) in the serum of WT and KO mice by ELISA. We found that there was no difference in the anti-NP_36_-IgM in serum from WT and KO mice (Fig. 6N). Whereas, the levels of anti-NP_2_-IgG1 and anti-NP_36_-IgG1 significantly decreased in the serum of KO mice compared to WT mice (Fig. 6O and 6P). Furthermore, the affinity of anti-NP-IgG1 antibodies (expressed as the ratio of NP_2_-IgG1/NP_36_-IgG1) was significantly lower than that in WT mice (Fig. 6Q). These results indicate that DDIT3 deficiency can attenuate antibody responses. What’s more, we further performed the BCR sequence and analyzed BCR cloning frequency. Here, we divided the clone frequencies into single, median, and large. We found single and median rosed slightly for IgH in KO mice (Fig. S8N). Meanwhile, there was no change in the cloning frequency for IgK and IgL (Fig. S8O and S8P). These results suggest that DDIT3 plays a critical role in humoral immune response and germinal center response.

### DDIT3 deficiency ameliorates lupus-like autoimmunity and renal injury in a cGVHD model

**Figure 7.**
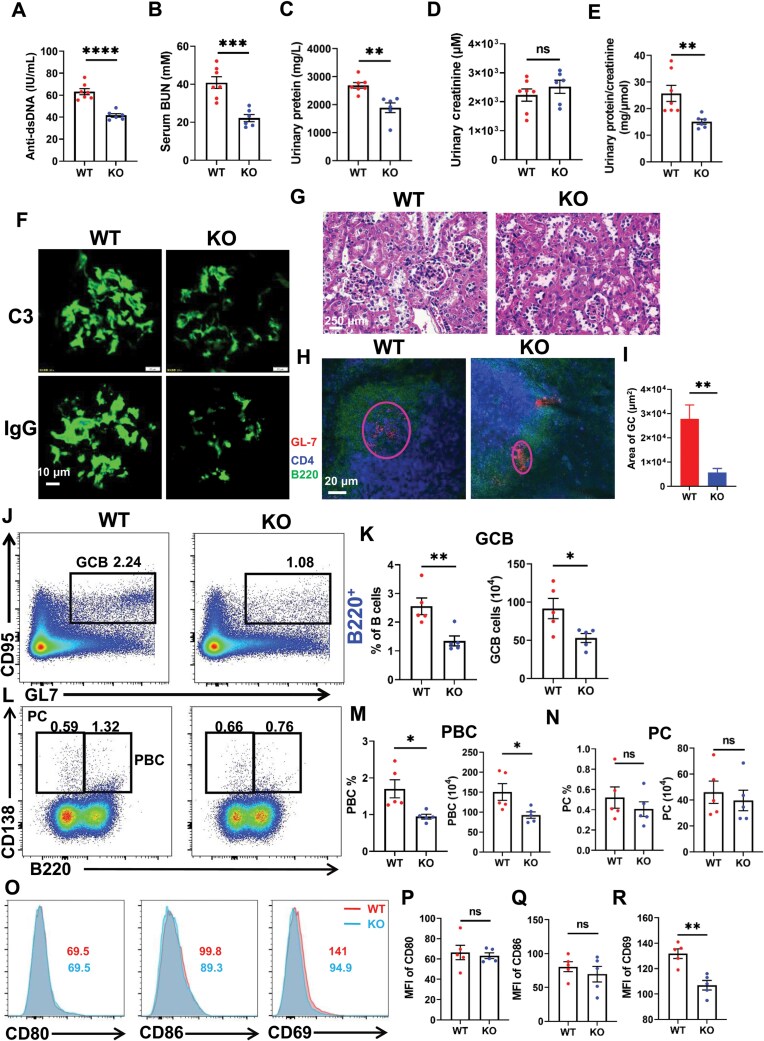
**DDIT3 deficiency ameliorates lupus-like autoimmunity and renal injury in a cGVHD model.** (A) ELISA analysis for anti-dsDNA antibody in serum from BM12 induced-WT (*n* = 7) and KO mice (*n* = 6) for 4 weeks. (B) Serum BUN in WT (*n* = 7) and KO (*n* = 6) mice. (C) BCA analysis of urinary protein in WT (*n* = 7) and KO (*n* = 6) mice. (D) Urinary creatinine in WT (*n* = 7) and KO (*n *= 6) mice. (E) Ratio of urinary protein to urinary creatinine in WT (*n* = 7) and KO (*n* = 6) mice. (F) Immunofluorescence staining of C3 and total IgG in kidneys from induced WT and KO mice. Scale bar, 10 μm. (G) H&E analysis of kidneys from WT and KO mice. Scale bar, 250 μm. (H) Immunofluorescence staining of GCs from WT and KO mice. Shown are representative GCs using anti-CD4 (blue), anti-B220 (green), and anti-GL-7 (red). Scale bar, 20 μm. (I) Quantification of the area of GCs from WT (*n* = 3) and KO (*n* = 3) mice. (J–N) Representative flow diagrams and statistical analysis of GCB, PBC, and PC in BM12 induced WT (*n* = 5) and KO (*n* = 5) mice. (O–R) Flow cytometry analysis of CD80, CD86, and CD69 expression in B cell from BM12-induced WT (*n* = 5) and KO mice (*n* = 5). ns = no significance, *P < 0.05; **P < 0.01; ***P < 0.001, ****P < 0.0001, unpaired student’s *t*-test.

To investigate the impact of DDIT3 on the development of SLE, we conducted a chronic graft-versus-host disease (cGVHD) lupus-like model by transferring CD4^+^ T cells from Bm12 mice into WT and KO mice [30]. Initially, we assessed the levels of anti-dsDNA antibodies and blood urea nitrogen (BUN) in the serum after Bm12 induction for 4 weeks, as well as urinary protein and urinary creatinine in both WT and KO mice, and discovered that anti-dsDNA antibodies (Fig. 7A), serum BUN (Fig. 7B), and urinary protein (Fig. 7C) were markedly reduced, but no change in urinary creatinine (Fig. 7D) in KO mice when compared to WT mice. The ratio of urine protein to urine creatinine is commonly used to estimate the 24-hour urine protein level in mice because random urinary protein tends to yield large errors. Our further analysis revealed that the ratio was significantly lower in KO mice than in WT mice (Fig. 7E). Subsequently, we examined the deposition of complement C3 and total IgG in the kidneys of WT and KO mice using confocal microscopy and found that less C3 and total IgG were deposited in the glomeruli of KO mice compared with WT mice (Figs. 7F, S9A and S9B). Additionally, compared with WT mice, less cell infiltration in the glomeruli and renal pathological amelioration were observed in KO mice (Figs. 7G and S9C). These results indicated that DDIT3 deficiency improves laboratory parameters and alleviates renal histological alterations.

Lastly, to further investigate whether the attenuated humoral immune response in KO mice is related to the pathogenesis of SLE, we examined the germinal centers and splenic lymphocytes using confocal microscopy and flow cytometry, respectively. Compared with WT mice, the germinal center area was smaller in the spleens of KO mice after Bm12 induction (Fig. 7H and 7I). Furthermore, the frequencies and numbers of T2 and MZ B cells (Fig. S9D–G), GCBs, and PBCs were significantly reduced in KO mice compared with WT mice (Fig. 7J–M). However, there were no significant differences observed in the frequencies and numbers of PCs (Fig. 7N), FO, IS, and T1 B cells, and follicular helper T cells (Tfh) cells between WT and KO mice (Fig. S9H–N). Additionally, we analyzed the expression of CD69, CD80, and CD86 as activation markers on splenic B cells, and observed that the MFI of CD69 was significantly diminished in KO B cells compared to WT B cells. However, there were no significant differences in the MFI of CD80 and CD86 between WT and KO mice (Fig. 7O–R). Furthermore, we detected the B cell activation, proliferation, and apoptosis *in vitro* upon LPS stimulation by flow cytometry. We found that both the proliferation and apoptosis in KO B cells were significantly decreased when compared to WT B cells (Fig. S9O and S9P). And the MFI of CD80 was significantly increased but CD86 and CD69 were significantly decreased in KO B cells when compared to WT B cells (Fig. S9Q and S9R). These data suggest that deficiency of DDIT3 ameliorates the development of SLE by inhibiting the germinal center response and B cell activation, thereby reducing autoantibodies and renal injury.

## Discussion

**Figure 8.**
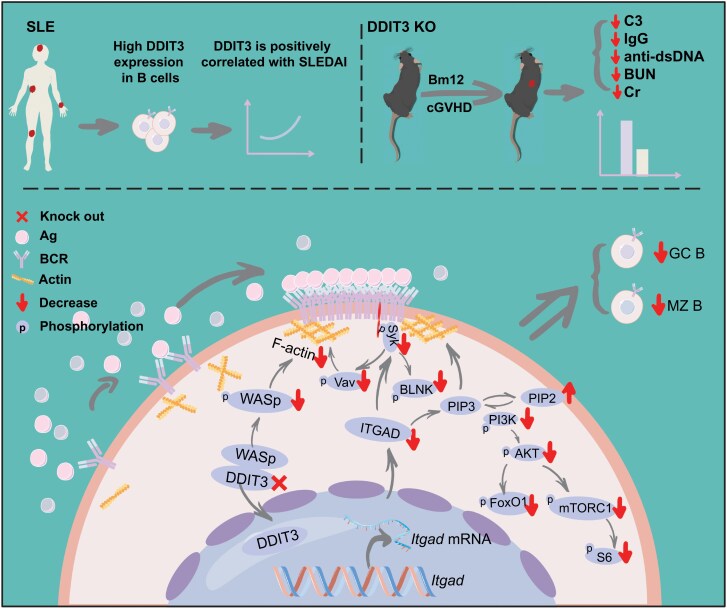
**DDIT3 deficiency ameliorates SLE by regulating B cell activation and differentiation.** DDIT3 expression is increased in B cells from SLE patients and positively correlated with disease activity. Mechanistically, DDIT3 promotes the *Itgad* transcription and WASp phosphorylation, which enhances actin polymerization, promoting B cell activation, BCR signaling, and B cell differentiation. Finally, DDIT3 deficiency attenuates lupus autoimmunity and disease symptoms in a cGVHD model.

DDIT3 has been reported to play a key role in host innate immunity and viral replication. DDIT3 enhances the replication of bovine viral diarrhea virus by inducing the expression of OTU deubiquitinating 1, which leads to the degradation of mitochondrial antiviral signaling and inhibits IFN-I production [19]. Additionally, DDIT3 promotes bovine viral diarrhea virus replication by inducing SQSTM1-mediated autophagy and promoting STING degradation, thereby inhibiting innate immunity [20]. However, the role of DDIT3 in adaptive immunity remains unknown. In this study, we discovered that DDIT3 deficiency disrupts B cell development and differentiation. Moreover, DDIT3 enhances B cell activation and BCR signaling by enhancing *Itgad* transcription and expression, which regulates the reorganization of the actin cytoskeleton and PI3K BCR signaling. Furthermore, using the NP-KLH immune model, we found that DDIT3 is crucial for humoral responses and GC responses. Deficiency of DDIT3 ameliorates lupus-like autoimmunity and renal injury by inhibiting the germinal center response and B cell activation (Fig. 8). Therefore, our study reveals for the first time the role and function of DDIT3 in B cells, humoral immunity, and SLE development.

DDR plays a crucial role in the development and functioning of the immune system. It is involved in the formation of antigen receptors, maturation and differentiation of lymphocytes, and production of antibodies [13, 14]. Recent studies have reported abnormal DDR in various autoimmune diseases such as SLE, multiple sclerosis, and rheumatoid arthritis [13, 31–34]. In SLE, dysfunctional B cell activity is based on DDR [15]. One important member of the DNA damage signaling pathway, DDIT3, is upregulated by DDR [16]. A recent study constructed an artificial neural network diagnostic model for cSLE and identified several relevant signature genes, including DDIT3. DDIT3 expression is significantly increased in cSLE compared to healthy controls, and it is correlated with memory B cells in cSLE [21]. However, the role of DDIT3 in B cells of SLE remains unknown. In our study, we observed a significant increase in DDIT3 expression in total, naïve, and unswitched memory B cells of adult SLE patients compared to healthy controls. Furthermore, DDIT3 expression was higher in active SLE patients with high SLEDAI than in inactive SLE patients with low SLEDAI. DDIT3 expression showed a positive correlation with SLEDAI in total B cells and subpopulations. These findings suggest that DDIT3 may be involved in the pathogenesis of SLE by affecting B cells. Additionally, DDIT3 could serve as an indicator of disease activity and a potential therapeutic target. Furthermore, DDIT3 is also correlated with other immune cells such as activated dendritic cells, neutrophils, macrophages, CD8^+^ T cells, and resting memory CD4^+^ T cells in cSLE [21]. Therefore, it is worth investigating whether DDIT3 contributes to the pathogenesis of SLE by affecting these immune cells. In our study, there were no differences in DDIT3 expression on CD4^+^ T cells, CD8^+^ T cells, Tregs, and monocytes between SLE patients and healthy controls. Besides, in our study, we found that loss of DDIT3 did not affect the development and differentiation of CD4^+^ and CD8^+^ T cells as well as subpopulations. These results indicated that DDIT3 participates in the pathogenesis of SLE by affecting B cells but other lymphocytes.

Beta2-integrins are leukocyte-specific and comprise four members: CD11a/CD18 (LFA-1, alphaLbeta2), CD11b/CD18 (Mac-1, alphaMbeta2), CD11c/CD18 (CR4, alphaXbeta2), and CD11d/CD18 (αDβ2, alphaDbeta2) [35]. These integrins play a role in leukocyte trafficking, immunological synapse formation, TLR signaling, TCR signaling, and BCR signaling [35]. ITGAD, also known as CD11d, is an alpha subunit of beta2-integrins and is highly expressed in myeloid cells, B cells, and NK cells. It has low expression on peripheral blood leukocytes and higher expression on γδ T cells compared to αβ T cells [36, 37]. ITGAD has been reported to affect T cell development and proliferation in response to staphylococcal enterotoxin [38]. However, the role of ITGAD in B cells has been less studied. In our study, we discovered that DDIT3 enhances ITGAD expression, thereby promoting both proximal and distal BCR signaling, particularly PI3K signaling. BCR signaling is crucial for B cell function, suggesting that ITGAD may have an impact on humoral immune responses. ITGAD-KO mice could serve as a valuable tool for further investigation. On the other hand, another member of beta2-integrins, CD11b/CD18, has been reported to inhibit BCR signaling to maintain autoreactive B cell tolerance [39], and CD11b is one of the strongest genetic risk factors for SLE [40, 41]. These results indicated the different roles of β2-integrins in SLE and B cells.

B cell hyper-activation is critical to autoimmune B cell differentiation and autoantibodies secretion in the pathogenesis of SLE. B cell activation is regulated by actin cytoskeleton reorganization, and β2-integrins were reported to promote actin polymerization by inducing PIP3 formation [26–28], and then activates downstream of Src family tyrosine kinases, Syk, and Vav in T cells and B cells [29]. Upon engagement with antigen (Ag), BCR microclusters aggregate, which is regulated by actin and PIP3. B cells from SLE exhibit stronger BCR microclusters and higher levels of PIP3. Additionally, they also display hyper-activated PI3K signaling compared to healthy controls [12, 42]. According to the aforementioned results, our study has discovered that DDIT3 facilitates the activation of PI3K, actin polymerization, B cell activation, and BCR signaling by positively regulating the expression of ITGAD. Interestingly, it has been reported that the hyper-activation of mTOR leads to the loss of MZ B cells [43, 44]. However, in our study, we observed a decrease in MZ B cells in DDIT3 KO mice, along with a decrease in mTOR activation. This contradiction suggests that DDIT3 may regulate the differentiation of MZ B cells through another, potentially more significant, molecular mechanism.

Another intriguing discovery is the significant increase of CD69 in KO B cells within our Bm12-induced cGVHD model. CD69 is classified as an early activation marker for lymphocytes. However, the mechanism by which DDIT3 regulates CD69 expression in the Bm12-induced cGVHD model has yet to be investigated.

Our study collectively demonstrates that DDIT3 plays a role in the pathogenesis of SLE by regulating the development and differentiation of B cells, the activation of B cells, BCR signaling, and humoral immunity response. Additionally, we discovered that DDIT3 deficiency could ameliorate the symptoms of SLE (Fig. 8), thereby presenting a novel therapeutic target for lupus-like autoimmune responses.

## Research limitations

Although DDIT3 has been shown to promote the development and progression of SLE by regulating B cell development and differentiation, BCR signaling, and humoral immune response, this study has some limitations in planning and design. First, the exact mechanism needs to be further elucidated. For example, in CD21 or CD23 B-cell-specific knockout DDIT3 mice, the effect of DDIT3 on the pathogenesis and development of SLE through the regulation of actin polymerization pathway mediating B-cell function is further verified. In addition, the overexpression of DDIT3 in SLE mouse models requires further exploration to explore its effects on tissues throughout the body at different stages of SLE.

## Methods

### Microarray data and analysis

Series matrix files of the dataset GSE72326, GSE110169, and GSE65391 were downloaded from Gene Expression Omnibus (GEO) database. The dataset GSE72326 contained 177 peripheral blood mononuclear cells (PBMCs), 157 from SLE patients, and 20 healthy controls. The dataset GSE110169 contained 159 PBMC, 82 from SLE patients, and 77 healthy controls. The dataset GSE65391 contained 953 PBMC, 667 from LN, 245 from SLE patients, and 41 from healthy controls. Use the R package to standardize the expression matrix and annotate genes, and extract the expression values of DDIT3 for plot analysis based on grouping.

### Patients and PBMCs collection

Forty-nine patients diagnosed with SLE, and twenty-seven healthy individuals of similar age were recruited from Shanxi Bethune Hospital in Taiyuan, China. Patients with malignancy, lymphoproliferative disease, active infection, or other autoimmune diseases were excluded from the study. The basic characteristics of patients and healthy controls are shown in Table S1. The disease activity of SLE was assessed using the SLE Disease Activity Index 2000 (SLEDAI-2K). Prior to participation, all individuals provided written informed consent, and the study was approved by the Ethics Committee of Shanxi Bethune Hospital (YXLL-2023-227). Peripheral blood mononuclear cells (PBMCs) were isolated using Ficoll according to the provided instructions and frozen to liquid nitrogen according to the cell cryopreservation procedure until specimen collection was completed.

### Mice

*Ddit3* ^−/−^ mice were generated using *Ddit3*^+/−^ mice, which were provided by Professor Wang Cong-yi from Huazhong University of Science and Technology, Wuhan, China. The mice used in the experiment were aged 8–12 weeks, and *Ddit3*^+/+^ mice were used as the WT control. CD45.1 mice were purchased from Cyagen Biosciences (Guangdong, China). Bm12 mice (B6(C)-H2-Ab1bm12/KhEgJ) were provided by Professor Zheng Fang from Huazhong University of Science and Technology, Wuhan, China. The mice were housed and fed in individually ventilated cages under specified pathogen-free conditions. All animal study protocols were approved by the Institutional Review Board of Shanxi Medical University (2023-SKY-L-N103).

### Cell isolation and purification of B cells

Splenic lymphocytes were isolated through density gradient centrifugation using Ficoll, while spleen B cells were purified via complement-dependent cytotoxicity that T cells were lysed using anti-CD90.2 and guinea pig complement. Monocytes/macrophages were then eliminated by adhering to T75 at 37°C for 1 hour.

B cells for RNA-sequencing and RT-qPCR were purified with mouse CD19 microbeads.

### Immunoblot analysis

Purified B cells were incubated with Biotin-SP-conjugated AffiniPure F(ab’)^2^ Fragment Goat Anti-Mouse IgG + IgM (H + L) (10 μg/mL, sAg) on ice for 30 min. Streptavidin (10 μg/mL) was added and incubated for an additional 10 min. The cells were then activated at 37°C for the indicated time. The cell lysates were separated by SDS–PAGE and transferred to a nitrocellulose membrane. After being blocked with 5% milk in TBST, the bands were probed with anti-phosphotyrosine, anti-pWASp, anti-pPI3K, anti-pBLNK, anti-pSyk, anti-pFoxO1, anti-FoxO1, anti-PI3K, anti-pS6, anti-S6, anti-pmTOR, anti-mTOR, anti-Syk, anti-BLNK, anti-pAkt, anti-Akt, anti-WASp, anti-pVav, and anti-ITGAD. β-actin was used as a loading control. The immunoreactive bands were captured using the ChemiDoc™XRS-imaging systems, and the intensity was quantified using the Image Lab software.

### Hematoxylin-eosin staining

Mouse spleens and kidneys were formalin-fixed, embedded, and sectioned as described previously [45], and tissues were visualized under a Thermostatic freezing microtome by hematoxylin-eosin staining. The evaluation of glomerulonephritis and perivascular cellular infiltration was carried out by two renal pathologists, using semi-quantitative criteria to assess the renal biopsies.

### Flow cytometry and phosflow

Human peripheral PBMC were labeled with PE-anti-CD14, APC-anti-CD25, PE-Cy7-anti-CD127, PB-anti-CD4, BV510-anti-CD8, PE-anti-CD27, APC-CD19, PE-Cy7-anti-CD24, BV510-anti-IgD, Fixable Viability Stain 620, PB-anti-CD38 to analyze Lymphocyte subsets. To perform intracellular staining for DDIT3, the cells were fixed and permeabilized using a fixation/penetration kit and labeled with anti-DDIT3 and Alexa Fluor™ 488-Goat anti-Rabbit.

BM cells were extracted from the tibia and femur of mice. Red blood cells were lysed using a red blood cell lysis buffer. BM cells were then stained with FITC-anti-B220, PE-anti-BP-1, 7-AAD, APC-anti-IgM, PE-Cy7-anti-CD43, PB-CD24, and BV510-CD127.

Splenic lymphocytes were labeled with PE-anti-CD95, AF647-anti-GL-7, FITC-Annexin V, PE-anti-CD21/CD35, Percp/Cy5.5-anti-CD23, PB-anti-IgD, BV510-anti-CD19, BV510-anti-CD45.2, PE-Cy7-anti-CD45.1, FITC-anti-CD80, PE-anti-CD86, Percp-anti-B220, APC-anti-CD69 and BV510-anti-CD138, BV421-anti-IgM, PE-anti-ICOS (CD278), Percp-anti-CD44, APC-anti-CD4, PE-Cy7-anti-PD-1, BV421-anti-CXCR5, FITC-anti-B220, APC-anti-IgM, 7-AAD, and PE-NP to analyze B cell peripheral differentiation. To perform intracellular staining for PE-Cy7-anti-Ki-67, the cells were fixed and permeabilized using a fixation/penetration kit.

To detect DDIT3 expression in bone marrow and spleen B-cell subsets of wild-type mice, cells were stained with specific antibodies B-cell subsets. After fixed and permeabilized using a fixation/penetration kit, cells were labeled with anti-DDIT3, and followed by DyLight 488-Goat anti-mouse.

Peritoneal cavity cells were stained with FITC-anti-CD19, PB-anti-CD1d, PE-anti-CD5, Percp/Cy5.5-anti-CD11b, APC-anti-IgM, and PB-anti-IgD.

The lymphocytes of spleens, thymus lymphocytes, and lymph nodes were labeled with PE-anti-CD8, FITC-Annexin V, APC-anti-CD25, BV510-anti-CD8, Fixable Viability Stain 620, Percp-anti-CD44, PB-anti-CD4 to analyze T cell peripheral differentiation. To perform intracellular staining for AF488-anti-Foxp3, the cells were fixed and permeabilized using a fixation/penetration kit.

To perform phosflow analysis, purified B cells were subjected to the following steps: firstly, they were stained with BV510-anti-CD19 at 4°C for 30 min. Then, the cells were incubated with sAg on ice for 30 min. Subsequently, the cells were activated at 37°C for the indicated time. After activation, the cells were fixed and permeabilized using a fixation/penetration kit. Next, the cells were labeled with ActinGreen 488 ReadyProbes™ and anti-pWASp. Finally, the cells were incubated with AF647-Goat anti-Rabbit IgG (H + L) secondary antibody.

The samples were analyzed using FACSCanto^TM^ Ⅱ Flow Cytometry, and data analysis was performed using the software FlowJo software.

### T cell stimulation *in vitro*

The lymphocytes were stimulated in the medium containing 750 ng/mL Ionomycin and 50 ng/mL phorbol 12-myristate 13-acetate. 1 h later, 1× Brefeldin A was added to prevent protein transport. After 4 h, the lymphocytes were stained with PB-anti-CD4 and BV510-anti-CD8. Then cells were fixed and permeabilized using a fixation/penetration kit, and stained with PE-anti-IL-17A, APC-anti-IL-4, and PE-anti-IFN-γ.

### Confocal microscopy analysis

Purified splenic B cells were incubated with Alexa Fluor 594-AffiniPure F(ab’)2 Fragment Goat Anti-Mouse IgG + IgM (H + L) on ice for 30 min, followed by activation at 37°C for the specified duration. The cells were then fixed with 4% paraformaldehyde and permeabilized with 0.05% saponin. They were subsequently stained with anti-DDIT3, anti-pY, ActinGreen 488 ReadyProbes™, anti-pSyk, anti-pBLNK, anti-pPI3K, anti-pAkt, anti-pS6, and anti-pWASp. Lastly, cells were incubated with AF405-Goat anti-Rabbit IgG (H + L), AF647-Goat anti-Rabbit IgG (H + L), and DyLight 488 Conjugated AffiniPure Goat Anti-Mouse IgG (H + L). Images were captured using confocal microscopy. Data were analyzed using FV31 ASW software.

### Bone marrow chimeras

CD45.1 mice were subjected to hematopoietic lethal irradiation, with a dose of 9 Gy. 4 h later, a mixture of BM cells from WT or KO with CD45.1 at a ratio of 1:1, totaling 5 × 10^6^ cells per mouse, was intravenously transferred into the irradiated CD45.1 mice. After 8 weeks, the chimeric mice were analyzed.

### Induction of lupus mouse model

Splenic lymphocytes were collected from female Bm12 mice. The lymphocytes, totaling 4 × 10^7^ cells per mouse, were then transferred intraperitoneally (i.p.) into female WT and DDIT3 KO mice aged 8–10 weeks. Four weeks later, serum, urine, and spleens were collected for analysis.

### T-cell-dependent immune model

According to the specification, a mixture of NP-KLH and adjuvant was prepared with a concentration of 0.2 mg/mL. Subsequently, WT and KO mice, aged 8–10 weeks, were subcutaneously injected with 200 μL of the mixture. After 28 days of the first immunization, the mice were re-immunized with the same dose. Finally, the spleens and serum were collected 33 days after the first immunization.

### Enzyme linked immunosorbent assay (ELISA)

The serum immunoglobulins were quantified using the ELISA method. To begin with, the plates were coated with 2 μg/mL NP_2_-BSA and NP_36_-BSA. After that, the plates were blocked using 10% bovine serum albumin BSA. The mouse serum was diluted according to the specified titers and added to the coated plates. Subsequently, HRP-IgG1 or HRP-IgM were added, followed by the addition of the substrate. The absorbance of OD450 was measured using a microplate reader. The levels of anti-dsDNA in the serum were determined using the mouse dsDNA-Ab ELISA KIT, following the provided instructions.

### Immunofluorescence analysis of spleen and kidney

After performing optical cutting temperature reagent embedding on the spleen and kidney samples of WT and *Ddit3* KO mice, the samples were cut into 5 mm sections. These sections were then fixed with acetone for 5 min and allowed to dry at room temperature. Next, the spleen and kidney sections were blocked using a solution containing 5% BSA and 10% Fc blocker. Afterwards, the sections were stained overnight at 4°C with the following antibodies: AF647-anti-GL7, PB-CD4, FITC-anti-CD45R/B220, DyLight 488 Conjugated AffiniPure Goat Anti-Mouse IgG (H + L), and AF488-anti-C3. To prevent fluorescence quenching, an anti-quenching agent was added. Confocal microscopy was used to capture images, and the data were analyzed using FV31 ASW software. The ImageJ software quantifies positive signals in glomeruli as mean optical density.

### DDIT3 transcript analysis

Eight transcripts of DDIT3 were compared in the National Center of Biotechnology Information (NCBI) database.

PBMCs of HC or patient are prepared as described previously. These cells split red and then lysed by RIPA. The lysates were detected by immunoblotting and were probed with anti-DDIT3.

### RNA-sequence

RNA extraction was performed using the Total RNA Extraction reagent following the manufacturer’s instructions. The extracted total RNA samples were then sent to Novogene Inc. for RNA sequencing. Subsequently, the sequencing was performed on the Illumina NovaSeq 6000 platform. The downstream differences were analyzed using DESeq2 software (1.20.0). GO enrichment and KEGG analysis of the differentially expressed genes were conducted using clusterProfiler software (3.8.1).

### Urea, creatinine, and urine protein measure

Urea and creatinine levels were quantified using the urea content test kit and creatinine content test kit, respectively, employing enzymatic and sarcosine oxidase methods. Furthermore, the BCA protein concentration assay kit was utilized to measure urine protein levels.

### qPCR analysis

Total RNA was extracted with Total RNA Extraction reagent, and cDNA was synthesized with Reverse Transcription Kit. mRNA expression was detected by SYBR Green Master Mix according to the manufacturer’s instructions. The primer sequences (Table S2) were synthesized by Sangon (China).

### ChIP-qPCR

ChIP was conducted using the SimpleChIPTM Enzymatic Chromatin IP Kit (Magnetic Beads) according to the manufacturer’s instructions. Subsequently, we immunoprecipitated the digested lysates with 1μg of anti-DDIT3, anti-Histone H3, and normal Rabbit IgG. The fragment of the *Itgad* promoter was amplified by qPCR. The qPCR products were verified by gel electrophoresis on a 2% agarose gel in TBE buffer. The primer sequences can be found in Table S2.

### Dual-luciferase reporter gene assay

HEK293T cells were cultured in 6-well plates and co-transfected with pGL3-mITGAD promoter-luciferase and pAd5-E1-CMV/pAd5-E1-CMV-mDDIT3 using lip3000 transfection reagents (L3000015, Invitrogen), pCMV-RL as calibration. After 48 h, Cells were collected and tested according to the Dual-Luciferase® Report Assay Systems Kit.

### Co-immunocoprecipitation

Puriﬁed splenic B cells (1 × 10^7^) were activated for 0 and 5 min using the same method as described above. Cells were lysed and were incubated with 1 μg of the anti-DDIT3 or Rabbit Control IgG for 2 h, was incubated with 20 μL rProtein-A/G Plus Magpoly Beads overnight. The beads were washed with TBS and the immunoprecipitates were eluted using SDS loading buffer and boiled. The samples were analyzed by WB. The bands were probed with anti-pWASp, anti-WASp and anti-DDIT3.

### B cell stimulation *in vitro*

2 × 10^6^ WT and KO purified B cells stimulated for 3 days with 10 μg/mL LPS. Percp-anti-B220, FITC-anti-CD80, PE-anti-CD86, APC-anti-CD69 were stained to detect the B cell activation by flow cytomertry.

### B cell proliferation and apoptosis *in vitro*

2 × 10^6^ WT and KO purified B cells were washed twice with PBS, and then Cell Proliferation Dye eFluor™ 450 was added at a concentration of 5 μM for labeling before stimulation for 3 days with LPS. FITC-anti-B220 and PE-Annexin V were stained to detect the B cell proliferation and apoptosis by flow cytometry.

### BCR-sequence and analysis

After 33 days of immunization, 1 × 10^7^ cells/sample were frozen with dry ice from spleens WT and DDIT3 KO mice and sent to the Singleron Inc. for bulk BCR-seq. The core methodology involves capturing mRNA, RT-PCR amplification, BCR enrichment and library construction. The resultant library can be sequenced using the Illumina sequencing platform Novaseq 6000, employing a PE150 strategy for sequencing. BCR clonotype assignment was performed using celescope (v2.1.0) bulk VDJ pipeline with the IMGT database as reference.

### Research ethics

Prior to participation, all individuals provided written informed consent, and the study was approved by the Ethics Committee of Shanxi Bethune Hospital (YXLL-2023-227). All animal study protocols were approved by the Institutional Review Board of Shanxi Medical University (2023-SKY-L-N103).

### Statistical analysis

Prism 9.0 software was used for data analysis. Unpaired two-tailed Student’s *t*-tests were performed to assess the statistical difference between the two groups when the data were normally distributed. Multiple comparisons were performed using one-way ANOVA. Data were represented as mean ± SEM (standard error of mean). The correlation analysis was performed by Pearson’s *r*-test. *P *< 0.05 was considered significant.

## Supplementary Material

lnaf009_suppl_Supplementary_Figures_S1-S9

lnaf009_suppl_Supplementary_Table_S1

lnaf009_suppl_Supplementary_Table_S2

lnaf009_suppl_Supplementary_Table_S3

## Data Availability

All data supporting this paper are present within the paper and/or the Supplementary Materials. The RNA sequencing datasets generated for this study can be found in NCBI GEO under the following accession number GSE252782.

## References

[1] Wakeland EK , Liu K, Graham RR, et al Delineating the genetic basis of systemic lupus erythematosus. Immunity 2001;15:397–408. 11567630 10.1016/s1074-7613(01)00201-1

[2] Tsokos GC. Systemic lupus erythematosus. N Engl J Med 2011;365:2110–21. 22129255 10.1056/NEJMra1100359

[3] Klinman DM , Shirai A, Ishigatsubo Y, et al Quantitation of IgM- and IgG-secreting B cells in the peripheral blood of patients with systemic lupus erythematosus. Arthritis Rheum 1991;34:1404–10. 1719987 10.1002/art.1780341110

[4] Liossis SN , Kovacs B, Dennis G, et al B cells from patients with systemic lupus erythematosus display abnormal antigen receptor-mediated early signal transduction events. J Clin Invest 1996;98:2549–57. 8958217 10.1172/JCI119073 PMC507712

[5] Hampe CS. B cell in autoimmune diseases. Scientifica (Cairo) 2012;2012:215308. 23807906 10.6064/2012/215308 PMC3692299

[6] Dogan I , Bertocci B, Vilmont V, et al Multiple layers of B cell memory with different effector functions. Nat Immunol 2009;10:1292–9. 19855380 10.1038/ni.1814

[7] Wu H , Deng Y, Feng Y, et al Epigenetic regulation in B-cell maturation and its dysregulation in autoimmunity. Cell Mol Immunol. 2018;15:676–84. 29375128 10.1038/cmi.2017.133 PMC6123482

[8] Domeier PP , Schell SL, Rahman ZS. Spontaneous germinal centers and autoimmunity. Autoimmunity. 2017;50:4–18. 28166685 10.1080/08916934.2017.1280671 PMC5669068

[9] Huck S , Le Corre R, Youinou P, et al Expression of B cell receptor-associated signaling molecules in human lupus. Autoimmunity. 2001;33:213–24. 11683380 10.3109/08916930109008048

[10] Wu XN , Ye YX, Niu JW, et al Defective PTEN regulation contributes to B cell hyperresponsiveness in systemic lupus erythematosus. Sci Transl Med 2014;6:246ra99. 10.1126/scitranslmed.3009131 25101889

[11] Ban T , Sato GR, Nishiyama A, et al Lyn kinase suppresses the transcriptional activity of IRF5 in the TLR-MyD88 pathway to restrain the development of autoimmunity. Immunity 2016;45:319–32. 27521268 10.1016/j.immuni.2016.07.015

[12] Bacalao MA , Satterthwaite AB. Recent advances in lupus B cell biology: PI3K, IFNgamma, and chromatin. Front Immunol 2020;11:615673. 33519824 10.3389/fimmu.2020.615673 PMC7841329

[13] McNally JP , Millen SH, Chaturvedi V, et al Manipulating DNA damage-response signaling for the treatment of immune-mediated diseases. Proc Natl Acad Sci USA 2017;114:E4782–91. 28533414 10.1073/pnas.1703683114 PMC5474825

[14] Bednarski JJ , Sleckman BP. At the intersection of DNA damage and immune responses. Nat Rev Immunol 2019;19:231–42. 30778174 10.1038/s41577-019-0135-6 PMC6438741

[15] Manolakou T , Nikolopoulos D, Gkikas D, et al ATR-mediated DNA damage responses underlie aberrant B cell activity in systemic lupus erythematosus. Sci Adv 2022;8:eabo5840. 36306362 10.1126/sciadv.abo5840 PMC9616496

[16] Jauhiainen A , Thomsen C, Strömbom L, et al Distinct cytoplasmic and nuclear functions of the stress induced protein DDIT3/CHOP/GADD153. PLoS One 2012;7:e33208. 22496745 10.1371/journal.pone.0033208 PMC3322118

[17] Hu H , Tian M, Ding C, et al The C/EBP homologous protein (CHOP) transcription factor functions in endoplasmic reticulum stress-induced apoptosis and microbial infection. Front Immunol 2018;9:3083. 30662442 10.3389/fimmu.2018.03083 PMC6328441

[18] Isobe T , Tange S, Tasaki H, et al Upregulation of CHOP participates in caspase activation and virus release in human astrovirus-infected cells. J Gen Virol 2019;100:778–92. 30912739 10.1099/jgv.0.001250

[19] Wang S , Hou P, Pan W, et al DDIT3 targets innate immunity via the DDIT3-OTUD1-MAVS pathway to promote bovine viral diarrhea virus replication. J Virol 2021;95:e02351–20. 33361422 10.1128/JVI.02351-20 PMC8094964

[20] Wang S , Ma X, Guo J, et al DDIT3 antagonizes innate immune response to promote bovine alphaherpesvirus 1 replication via the DDIT3-SQSTM1-STING pathway. Virulence. 2022;13:514–29. 35259065 10.1080/21505594.2022.2044667 PMC8920142

[21] Wu J , Yang W, Li H. An artificial neural network model based on autophagy-related genes in childhood systemic lupus erythematosus. Hereditas 2022;159:34. 36114579 10.1186/s41065-022-00248-7 PMC9479435

[22] Milne CD , Paige CJ. IL-7: a key regulator of B lymphopoiesis. Semin Immunol 2006;18:20–30. 16303314 10.1016/j.smim.2005.10.003

[23] Zhu X , Hart R, Chang MS, et al Analysis of the major patterns of B cell gene expression changes in response to short-term stimulation with 33 single ligands. J Immunol 2004;173:7141–9. 15585835 10.4049/jimmunol.173.12.7141

[24] Jing Y , Dai X, Yang L, et al STING couples with PI3K to regulate actin reorganization during BCR activation. Sci Adv 2020;6:eaax9455. 32494627 10.1126/sciadv.aax9455 PMC7176427

[25] Jing Y , Kang D, Liu L, et al Dedicator of cytokinesis protein 2 couples with lymphoid enhancer-binding factor 1 to regulate expression of CD21 and B-cell differentiation. J Allergy Clin Immunol 2019;144:1377–90.e4. 31405607 10.1016/j.jaci.2019.05.041

[26] Axelsson L , Hellberg C, Melander F, et al Clustering of beta(2)-integrins on human neutrophils activates dual signaling pathways to PtdIns 3-kinase. Exp Cell Res 2000;256:257–63. 10739672 10.1006/excr.2000.4816

[27] Lofgren R , Ng-Sikorski J, Sjolander A, et al Beta 2 integrin engagement triggers actin polymerization and phosphatidylinositol trisphosphate formation in non-adherent human neutrophils. J Cell Biol 1993;123:1597–605. 7504676 10.1083/jcb.123.6.1597 PMC2290867

[28] Dib K. BETA 2 integrin signaling in leukocytes. Front Biosci 2000;5:D438–51. 10762594 10.2741/pathology

[29] Deckert M , Tartare-Deckert S, Couture C, et al Functional and physical interactions of Syk family kinases with the Vav proto-oncogene product. Immunity 1996;5:591–604. 8986718 10.1016/s1074-7613(00)80273-3

[30] Klarquist J , Janssen EM. The bm12 inducible model of systemic lupus erythematosus (SLE) in C57BL/6 mice. J Visualized Exp. 2015;1:e53319. 10.3791/53319 PMC4692688 26554458

[31] Davies RC , Pettijohn K, Fike F, et al Defective DNA double-strand break repair in pediatric systemic lupus erythematosus. Arthritis Rheum 2012;64:568–78. 21905016 10.1002/art.33334

[32] Alissafi T , Kalafati L, Lazari M, et al Mitochondrial oxidative damage underlies regulatory T cell defects in autoimmunity. Cell Metab 2020;32:591–604.e7. 32738205 10.1016/j.cmet.2020.07.001 PMC7611060

[33] Souliotis VL , Vougas K, Gorgoulis VG, et al Defective DNA repair and chromatin organization in patients with quiescent systemic lupus erythematosus. Arthritis Res Ther 2016;18:182. 27492607 10.1186/s13075-016-1081-3 PMC4973109

[34] Mensah KA , Chen JW, Schickel JN, et al Impaired ATM activation in B cells is associated with bone resorption in rheumatoid arthritis. Sci Transl Med 2019;11:eaaw4626. 31748230 10.1126/scitranslmed.aaw4626 PMC7167286

[35] Fagerholm SC , Guenther C, Llort Asens M, et al Beta2-integrins and interacting proteins in leukocyte trafficking, immune suppression, and immunodeficiency disease. Front Immunol 2019;10:254. 30837997 10.3389/fimmu.2019.00254 PMC6389632

[36] Van der Vieren M , Le Trong H, Wood CL, et al A novel leukointegrin, alpha d beta 2, binds preferentially to ICAM-3. Immunity 1995;3:683–90. 8777714 10.1016/1074-7613(95)90058-6

[37] Siegers GM , Barreira CR, Postovit LM, et al CD11d beta2 integrin expression on human NK, B, and gammadelta T cells. J Leukoc Biol 2017;101:1029–35. 27881604 10.1189/jlb.3AB0716-326RR

[38] Wu H , Rodgers JR, Perrard XY, et al Deficiency of CD11b or CD11d results in reduced staphylococcal enterotoxin-induced T cell response and T cell phenotypic changes. J Immunol 2004;173:297–306. 15210787 10.4049/jimmunol.173.1.297

[39] Ding C , Ma Y, Chen X, et al Integrin CD11b negatively regulates BCR signalling to maintain autoreactive B cell tolerance. Nat Commun 2013;4:2813. 24264377 10.1038/ncomms3813

[40] Fagerholm SC , MacPherson M, James MJ, et al The CD11b-integrin (ITGAM) and systemic lupus erythematosus. Lupus 2013;22:657–63. 23753600 10.1177/0961203313491851

[41] Faridi MH , Khan SQ, Zhao W, et al CD11b activation suppresses TLR-dependent inflammation and autoimmunity in systemic lupus erythematosus. J Clin Invest 2017;127:1271–83. 28263189 10.1172/JCI88442 PMC5373862

[42] Wang J , Xu L, Shaheen S, et al Growth of B cell receptor microclusters is regulated by PIP2 and PIP3 equilibrium and Dock2 recruitment and activation. Cell Rep. 2017;21:2541–57. 29186690 10.1016/j.celrep.2017.10.117

[43] Meena NK , Pattanayak SP, Ben-Nun Y, et al mTORC1 activation in B cells confers impairment of marginal zone microarchitecture by exaggerating cathepsin activity. Immunology 2018;155:505–18. 30144045 10.1111/imm.12996 PMC6231019

[44] Benhamron S , Tirosh B. Direct activation of mTOR in B lymphocytes confers impairment in B-cell maturation andloss of marginal zone B cells. Eur J Immunol 2011;41:2390–6. 21674478 10.1002/eji.201041336

[45] Shu H , Zhao H, Shi Y, et al Transcriptomics-based analysis of the mechanism by which Wang-Bi capsule alleviates joint destruction in rats with collagen-induced arthritis. Chin Med. 2021;16:31. 33845855 10.1186/s13020-021-00439-w PMC8042720

