# Supplementary material for: DDIT3 deficiency ameliorates systemic lupus erythematosus by regulating B cell activation and differentiation
Source: Life Med. 2025 Mar 3;4(1):lnaf009. doi: 10.1093/lifemedi/lnaf009 (PMC11956853; doi:10.1093/lifemedi/lnaf009)
Supplement: lnaf009_suppl_Supplementary_Figures_S1-S9 [file lnaf009_suppl_supplementary_figures_s1-s9.docx]

**DDIT3 deficiency** **ameliorates systemic lupus erythematosus by regulating B cell activation and differentiation**

Xin Dai^1,#,*^, Jiali Yu^2,#^, Yunfei Zhang^3^, Zhiming Wang^4^, Yunyan Dai^4^, Ying Hu^3^, Xiaocui Wang^3^, Binbin Tian^5^, Minhui Wu^1^, Hao Chen^6^, Ruigao Song^7^, Dan Ma^8^, Cong-yi Wang^9^, Dawei Ye^10^, Ziliang Zheng^11,*^, Liyun Zhang^8,*^, Jing Luo^2,*^, Yukai Jing^12,^^13,*^

^1^Institute of Clinical Medicine, Central People's Hospital of Zhanjiang, Zhanjiang 524045, China

^2^Department of Rheumatology, The Second Hospital of Shanxi Medical University, Taiyuan 030009, China

^3^Department of Clinical Laboratory, Third Hospital of Shanxi Medical University, Shanxi Bethune Hospital, Shanxi Academy of Medical Sciences, Tongji Shanxi Hospital, Taiyuan 040030, China

^4^Department of General Surgery, Third Hospital of Shanxi Medical University, Shanxi Bethune Hospital, Shanxi Academy of Medical Sciences, Tongji Shanxi Hospital, Taiyuan 040030, China

^5^Department of Critical Care Medicine, Central People's Hospital of Zhanjiang, Zhanjiang, Guangdong 524045, China

^6^Key Laboratory of Biomedical Information Engineering of Ministry of Education, Biomedical Informatics & Genomics Center, School of Life Science and Technology, Xi’an Jiaotong University, Xi’an 710049, China

^7^Center of Reproductive Medicine, Shanxi Bethune Hospital, Shanxi Academy of Medical Sciences, Tongji Shanxi Hospital, Third Hospital of Shanxi Medical University, Taiyuan 030032, China

^8^Department of Rheumatology, Shanxi Bethune Hospital, Shanxi Academy of Medical Sciences, Tongji Shanxi Hospital, Third Hospital of Shanxi Medical University, Taiyuan 030032, China

^9^Department of Respiratory and Critical Care Medicine, the Center for Biomedical Research, NHC Key Laboratory of Respiratory Diseases, Tongji Hospital, Tongji Medical College, Huazhong University of Science and Technology, Wuhan 430030, China

^10^Cancer Center, Tongji Hospital, Tongji Medical College, Huazhong University of Science and Technology, Wuhan 430030, China

^11^Laboratory of Molecular Imaging, Fifth hospital of Shanxi Medical University (Shanxi Provincial People’s Hospital), Taiyuan 030032, China

^12^Department of Clinical Laboratory, Shanxi Province Clinical Research Center for Dermatologic and Immunologic Diseases (Rheumatic diseases), Shanxi Bethune Hospital, Shanxi Academy of Medical Sciences, Tongji Shanxi Hospital, Third Hospital of Shanxi Medical University, Taiyuan 030032, China

^13^Shanxi Academy of Advanced Research and Innovation, Taiyuan 030032, China

^#^These authors contributed equally to this work.

^*^Correspondence: jyk238507@sxmu.edu.cn (Y.J.), ljty966@hotmail.com (J.L.), zhangly@sxbqeh.com.cn (L.Z.), daixin415@126.com (X.D.), zzlsxty@sxmu.edu.cn (Z.Z.)

**Figure S1.** **Gating strategies of T-cell subsets, monocytes, and B-cell subsets in PBMC from SLE patients and HC.**

**Figure S2. Identification of DDIT3 transcription subtype in SLE patients and HC.**

(A) DDIT3 transcript comparison in NCBI database. (B) Immunoblot analysis was performed to identify and analyze the DDIT3 expression in HC (*n* = 4) and SLE patients (*n* = 4). (C) Statistical analysis of relative intensity of DDIT3/β-actin, **P* < 0.05, unpaired student’s *t*-test.

**Figure S3.** **Identification of ddit3 knockout mice.**

(A) PCR identification analysis of the DDIT3 alleles. Expected fragment size: −/− = 360 bp, +/− = 360 bp and 241 bp, +/+ = 241 bp. (B) Immunoblot analysis was performed to identify and analyze the DDIT3 expression in splenic lymphocytes from WT and KO mice. (C) Representative flow diagrams of DDIT3 expression of B cell subpopulations in spleen and bone marrows.

**Figure S4. DDIT3 deficiency disrupts the development and differentiation of B cells.**

(A–E) Representative flow diagrams and statistical analysis of follicular (FO), transitional 1 (T1), transitional 2 (T2), and IS B cells in spleens from WT (*n* = 6) and KO mice (*n* = 6). (F, G) Statistical analysis of apoptosis (Annexin V) and proliferation (Ki-67) for total, FO, MZ, T1, T2, and IS B cells in spleens from WT (*n* = 6) and KO mice (*n* = 6). Shown are the proportions and MFI of Annexin V and Ki-67. (H–J) Representative flow diagrams and statistical analysis of peritoneal B1a and B1b in WT (*n* = 6) and KO mice (*n* = 6). (K) Statistical analysis of splenic FO, T1, T2, and IS B cells in CD45.2^+^ and CD45.1^+^ cells from chimera mice (*n* = 5). ns = no significance, unpaired student’s *t*-test.

**Figure S5.** **DDIT3 deficiency** **did not affect T cell development and differentiation.**

(A) Gating strategy for splenic T cell subpopulations. (B–H) Statistical analysis of CD4^+^ T, CD8^+^ T, CD4^+^Foxp3^+^ (Treg), CD44^+^IFNγ^+^CD4^+^ (Th1), CD44^+^IL4^+^CD4^+^ (Th2), CD44^+^CD17^+^CD4^+^ (Th17), and CD44^+^IL4^+^CD8^+^ (CTL) in spleens, thymus, and lymph nodes from WT (*n* = 3) and KO mice (*n* = 3). ns = no significance, unpaired student’s *t*-test.

**Figure S6.** **DDIT3 positively regulates the B cell activation and BCR signaling.**

Splenic B cells from WT and DDIT3 KO mice were stimulated with AF594-anti-F(ab’)^2^ goat anti-mouse IgG + IgM (10 μg/mL) for indicated time, and the BCR signaling was analyzed by confocal. (A–D) Confocal analysis of pSyk and pPI3K in splenic B cells from WT and DDIT3 KO mice. Representative picture and Pearson correlation coefficients between BCR and pSyk, BCR and pPI3K. Scale bar, 2 μm. (E–K) Statistical analysis of relative intensity of pBLNK/BLNK, pSyk/Syk, pPI3K/PI3K, pAkt1/Akt1, pFoxO1/FoxO1, pmTOR/mTOR, and pS6/ S6 from three independent WB experiments. **P* < 0.05; ***P* < 0.01, unpaired student’s *t*-test.

**Figure S7.** **RNA-seq analysis of WT and KO B cells.**

(A) GO analysis of significantly different genes. (B–C) RT-qPCR analysis of the expression of *Dock1* and *Itga7* in B cells from WT mice and KO mice (*n* = 3). ns = no significant difference, unpaired student’s *t*-test.

**Figure S8.** **DDIT3 deficiency reduces the T-dependent immune response.**

(A–C) Statistical analysis of T1, FO, and T2 B cells from NP-KLH immunized WT and KO mice (*n* = 4). (D–J) Representative flow diagrams and statistical analysis of PC, PBC, CXCR5^+^ICOS^hi^ Tfh, and CXCR5^+^PD-1^hi^ Tfh from NP-KLH immunized WT and KO mice (*n* = 4). (K) Statistical analysis of CD4^+^ T from NP-KLH immunized WT and KO mice (*n* = 4). (L–M) Representative flow diagrams and statistical analysis of NP^+^GCB from NP-KLH immunized WT and KO mice (*n* = 4). (N–P) Clone frequency of BCR are defined as IgH (N), IgK (O), IgL (P) for WT and DDIT3 KO mice, Single, Freq = 1; Median, Freq > 1 & Freq ≤ 10; Large, Freq > 10. ns = no significant difference, **P* < 0.05, unpaired student’s *t*-test.

**Figure S9.** **DDIT3 deficiency ameliorates lupus-like symptoms in a cGVHD model.**

(A–C) Statistical analysis for immunofluorescence staining of C3 and total IgG in kidneys, and renal injury scores between WT (*n* = 5) and KO (*n* = 5) mice. (D–J) Representative flow diagrams and statistical analysis of MZ, T2, FO, T1 and IS B cells from BM12 induced-WT (*n* = 5) and KO (*n* = 5) mice. (K–N) Representative flow diagrams and statistical analysis of CXCR5^+^ICOS^hi^ Tfh, and CXCR5^+^PD-1^hi^ Tfh from BM12 induced-WT (*n* = 5) and KO mice (*n* = 5). (O–P) Representative flow diagrams and statistical analysis of proliferation (O) and Annexin V (P) of spleen B cells stimulated by LPS in WT (*n* = 3) and KO mice (*n* = 3). (Q–R) Representative flow diagrams and statistical analysis of CD80, CD86, and CD69 expression of spleen B cells stimulated by LPS in WT (*n* = 3) and KO (*n* = 3) mice. ns = no significant difference, **P* < 0.05, ***P* < 0.01, *****P* < 0.0001, unpaired student’s *t*-test.

**Table S1.** **Basic characteristic of SLE patients and healthy controls.**

**Table S2.** **Primers for PCR, RT-qPCR and CHIP-qPCR.**

**Table S3. Differentially expressed genes in splenic B cells from WT and DDIT3 KO mice.**

**Table S4. Information on reagents or resources.**
