# Supplementary material for: DDIT3 deficiency ameliorates systemic lupus erythematosus by regulating B cell activation and differentiation
Source: Life Med. 2025 Mar 3;4(1):lnaf009. doi: 10.1093/lifemedi/lnaf009 (PMC11956853; doi:10.1093/lifemedi/lnaf009)
Supplement: lnaf009_suppl_Supplementary_Table_S1 [file lnaf009_suppl_supplementary_table_s1.docx]

**Table S1. Upregulated and Downregulated Gene Information from RNA-seq Sequencing**

| Gene Symbol | P-value | Fold Change | Gene Title |
| --- | --- | --- | --- |
| Upregulated Genes |  |  |  |
| Col5a3 | 7.36E-26 | 3.269669544 | Collagen Type V Alpha 3 Chain |
| Myadm | 4.99E-22 | 1.298378797 | Myeloid Associated Differentiation Marker |
| Ddx3y | 4.05E-21 | 12.19674848 | DEAD-Box Helicase 3 Y-Linked |
| Eif2s3y | 1.11E-20 | 12.0501094 | Eukaryotic Translation Initiation Factor 2, Subunit 3, Structural Gene Y-linked |
| Kdm5d | 1.66E-19 | 11.73485751 | Lysine Demethylase 5D |
| Uty | 7.46E-15 | 10.36016954 | Ubiquitously Transcribed Tetratricopeptide Repeat Containing, Y-Linked |
| Rag1 | 1.55E-14 | 3.040575551 | Recombination Activating 1 |
| Gas7 | 5.27E-09 | 2.237334901 | Growth Arrest Specific 7 |
| Lmna | 7.51E-07 | 2.027552913 | Lamin A/C |
| Lcn2 | 4.54E-06 | 2.4632411285 | Lipocalin 2 |
| Prkcg | 1.26E-05 | 1.50519413 | Protein Kinase C Gamma |
| 2900026A02Rik | 0.000298647 | 1.730873331 |  |
| Rapgefl1 | 0.000429739 | 1.658706776 | Rap Guanine Nucleotide Exchange Factor Like 1 |
| Crip2 | 0.000449371 | 1.440650841 | Cysteine Rich Protein 2 |
| Islr2 | 0.000537162 | 1.387391789 | Immunoglobulin Superfamily Containing Leucine Rich Repeat 2 |
| Hspg2 | 0.001262582 | 1.418715688 | Heparan Sulfate Proteoglycan 2 |
| Adgrl2 | 0.013710407 | 1.533447666 | Adhesion G Protein-Coupled Receptor L2 |
| Tal1 | 0.013754864 | 4.321195566 | TAL BHLH Transcription Factor 1, Erythroid Differentiation Factor |
| Itga7 | 0.015682013 | 4.516728432 | Integrin Subunit Alpha 7 |
| Nuak1 | 0.026418987 | 1.015688984 | NUAK Family Kinase 1 |
| Cd177 | 0.029373023 | 2.298098936 | CD177 Molecule |
| Phlda3 | 0.031371534 | 3.191431996 | Pleckstrin Homology Like Domain Family A Member 3 |
| Mid1 | 0.032661431 | 1.417797434 | Midline 1 |
| Dock1 | 0.033380646 | 3.805240708 | Dedicator Of Cytokinesis 1 |
| BC030867 | 0.03372131 | 1.870713307 |  |
| Steap4 | 0.041683451 | 1.397747303 | STEAP4 Metalloreductase |
| Nptxr | 0.041979532 | 2.883989561 | Neuronal Pentraxin Receptor |
| Gfra1 | 0.044611503 | 2.217394063 | GDNF Family Receptor Alpha 1 |
| Bst1 | 0.045369579 | 1.22859585 | Bone Marrow Stromal Cell Antigen 1 |
| Rhoc | 0.045471435 | 2.822823301 | Ras Homolog Family Member C |
| Downregulated Genes | | | |
| Slc40a1 | 6.27E-19 | -2.05985804 | Solute Carrier Family 40 Member 1 |
| C1qc | 8.31E-19 | -2.240359803 | Complement C1q C Chain |
| C1qb | 2.53E-17 | -1.96307035 | Complement C1q B Chain |
| Vcam1 | 4.58E-16 | -2.046254772 | Vascular Cell Adhesion Molecule 1 |
| Axl | 2.26E-15 | -1.691126467 | AXL Receptor Tyrosine Kinase |
| Fcna | 8.41E-13 | -1.923560142 | Ficolin 3 |
| Cd5l | 2.87E-11 | -2.174056407 | CD5 Molecule Like |
| Csf1r | 3.18E-11 | -1.372961298 | Colony Stimulating Factor 1 Receptor |
| C1qa | 1.40E-10 | -1.79414251 | Complement C1q A Chain |
| Ddit3 | 7.32E-09 | -1.334542544 | DNA Damage Inducible Transcript 3 |
| Hmox1 | 5.27E-06 | -1.272020515 | Heme Oxygenase 1 |
| Itgad | 2.12E-05 | -1.714075314 | Integrin Subunit Alpha D |
| Mrc1 | 3.80E-05 | -1.606085367 | Mannose Receptor C-Type 1 |
| Adgre4 | 8.18E-05 | -1.757542205 | Adhesion G Protein-Coupled Receptor E4 |
| Slc11a1 | 0.000169611 | -1.86279535 | Solute Carrier Family 11 Member 1 |
| Hebp1 | 0.0013071 | -2.396634161 | Heme Binding Protein 1 |
| Clec4n | 0.002031971 | -1.886160454 | C-Type Lectin Domain Containing 4N |
| Slpi | 0.00587898 | -1.271086017 | Secretory Leukocyte Peptidase Inhibitor |
| Treml4 | 0.008111809 | -1.623629672 | Triggering Receptor Expressed On Myeloid Cells Like 4 |
| Cadm1 | 0.008307169 | -2.042408914 | Cell Adhesion Molecule 1 |
| Spic | 0.009614628 | -1.376315137 | Spi-C Transcription Factor |
| Hpgd | 0.009614628 | -1.385226708 | 15-Hydroxyprostaglandin Dehydrogenase |
| Tlr3 | 0.015682013 | -1.092927153 | Toll Like Receptor 3 |
| Mafb | 0.021614212 | -1.382885905 | MAF BZIP Transcription Factor B |
| Ptgis | 0.042356505 | -3.598939625 | Prostaglandin I2 Synthase |
| Kcnj10 | 0.042621107 | -1.919631587 | Potassium Inwardly Rectifying Channel Subfamily J Member 10 |
| Adamdec1 | 0.045471435 | -1.671808832 | ADAM Like Decysin 1 |
| Cd163 | 0.048133738 | -1.608047949 | CD163 Molecule |
| Ccdc88a | 0.048133738 | -1.266536132 | Coiled-Coil Domain Containing 88A |
| Slco2b1 | 0.048133738 | -2.326300472 | Solute Carrier Organic Anion Transporter Family Member 2B1 |
|  |  |  |  |
