# Supplementary material for: DDIT3 deficiency ameliorates systemic lupus erythematosus by regulating B cell activation and differentiation
Source: Life Med. 2025 Mar 3;4(1):lnaf009. doi: 10.1093/lifemedi/lnaf009 (PMC11956853; doi:10.1093/lifemedi/lnaf009)
Supplement: lnaf009_suppl_Supplementary_Table_S2 [file lnaf009_suppl_supplementary_table_s2.docx]

**Table S2. Basic characteristics of SLE patients and healthy controls.**

| Sample | age | gender | SLEADI-2k | Sample | age | gender | SLEADI-2k |
| --- | --- | --- | --- | --- | --- | --- | --- |
| Patient 1 | 51 | Female | 16 | Patient 39 | 56 | Female | 5 |
| Patient 2 | 28 | Female | 2 | Patient 40 | 50 | Female | 13 |
| Patient 3 | 49 | Female | 4 | Patient 41 | 20 | Male | 19 |
| Patient 4 | 50 | Female | 6 | Patient 42 | 32 | Female | 17 |
| Patient 5 | 21 | Female | 18 | Patient 43 | 17 | Female | 6 |
| Patient 6 | 40 | Female | 8 | Patient 44 | 33 | Female | 15 |
| Patient 7 | 58 | Female | 10 | Patient 45 | 38 | Female | 6 |
| Patient 8 | 39 | Female | 12 | Patient 46 | 26 | Male | 4 |
| Patient 9 | 26 | Female | 6 | Patient 47 | 28 | Female | 4 |
| Patient 10 | 39 | Female | 19 | Patient 48 | 23 | Female | 4 |
| Patient 11 | 28 | Male | 4 | Patient 49 | 28 | Female | 8 |
| Patient 12 | 28 | Female | 1 | HC 1 | 34 | Female |  |
| Patient 13 | 27 | Male | 17 | HC 2 | 41 | Female |  |
| Patient 14 | 29 | Female | 11 | HC 3 | 28 | Male |  |
| Patient 15 | 35 | Female | 22 | HC 4 | 26 | Male |  |
| Patient 16 | 25 | Female | 4 | HC 5 | 23 | Female |  |
| Patient 17 | 33 | Male | 13 | HC 6 | 36 | Male |  |
| Patient 18 | 34 | Female | 6 | HC 7 | 38 | Male |  |
| Patient 19 | 15 | Female | 10 | HC 8 | 31 | Female |  |
| Patient 20 | 47 | Female | 12 | HC 9 | 30 | Female |  |
| Patient 21 | 40 | Female | 12 | HC 10 | 31 | Female |  |
| Patient 22 | 34 | Male | 5 | HC 11 | 30 | Female |  |
| Patient 23 | 27 | Female | 4 | HC 12 | 38 | Female |  |
| Patient 24 | 22 | Female | 7 | HC 13 | 28 | Male |  |
| Patient 25 | 51 | Female | 6 | HC 14 | 37 | Female |  |
| Patient 26 | 47 | Female | 8 | HC 15 | 33 | Female |  |
| Patient 27 | 15 | Male | 18 | HC 16 | 32 | Male |  |
| Patient 28 | 22 | Female | 5 | HC 17 | 40 | Male |  |
| Patient 29 | 56 | Female | 4 | HC 18 | 45 | Female |  |
| Patient 30 | 41 | Female | 3 | HC 19 | 38 | Female |  |
| Patient 31 | 33 | Female | 4 | HC 20 | 33 | Female |  |
| Patient 32 | 43 | Female | 7 | HC 21 | 30 | Female |  |
| Patient 33 | 55 | Female | 8 | HC 22 | 40 | Male |  |
| Patient 34 | 39 | Male | 6 | HC 23 | 27 | Female |  |
| Patient 35 | 24 | Female | 18 | HC 24 | 46 | Female |  |
| Patient 36 | 64 | Female | 6 | HC 25 | 44 | Male |  |
| Patient 37 | 47 | Male | 1 | HC 26 | 28 | Female |  |
| Patient 38 | 30 | Female | 12 | HC 27 | 28 | Male |  |
