# Supplementary material for: DDIT3 deficiency ameliorates systemic lupus erythematosus by regulating B cell activation and differentiation
Source: Life Med. 2025 Mar 3;4(1):lnaf009. doi: 10.1093/lifemedi/lnaf009 (PMC11956853; doi:10.1093/lifemedi/lnaf009)
Supplement: lnaf009_suppl_Supplementary_Table_S3 [file lnaf009_suppl_supplementary_table_s3.docx]

| **Name** | **Forward primer (5’-3’)** | **Reverse primer (5’-3’)** |
| --- | --- | --- |
| *Itgad* RT-qPCR | CTGAGAGCCCAGGTGTCAAC | GTGGAGGAGTCTCATGGTTGG |
| *Itga7* RT-qPCR | TTGCTCGCTGGACTGTTCTT | CCACCAGCAGCCAGCTC |
| *Dock1* RT-qPCR | GGCCAGGCTCTGATGGATTT | GTCAGCGGGCTTGAATTCCT |
| *Gapdh* RT-qPCR | GGTGAAGGTCGGTGTGAACG | CTCGCTCCTGGAAGATGGTG |
| *ITGAD* RT-qPCR | CCATGATCAGCAGGCAGGAA | AGTCAGAATGCTGGGGAGGT |
| *GAPDH* RT-qPCR | GCAAATTCCATGGCACCGT | GCCCCACTTGATTTTGGAGG |
| *Itgad* ChIP-qPCR | CAGAAGTTCACCGACCCACA | TGTGCATGATCCCCTTGCAG |
| *Ddit3* PCR-WT | CAGATCCTCATACCAGGCTTCC | CTCCACCCTCTGCCAATGTAG |
| *Ddit3* PCR-KO | GCCAGGGTTTTCCCAGTCAC | CTCCACCCTCTGCCAATGTAG |

**Table S3. Sequences of primers for PCR, RT-qPCR and CHIP-qPCR.**
